# Seasonal variation of genotypes and reproductive plasticity in a facultative clonal freshwater invertebrate animal (*Hydra oligactis*) living in a temperate lake

**DOI:** 10.1002/ece3.9096

**Published:** 2022-07-14

**Authors:** Máté Miklós, Levente Laczkó, Gábor Sramkó, Zoltán Barta, Jácint Tökölyi

**Affiliations:** ^1^ MTA‐DE “Momentum” Ecology, Evolution and Developmental Biology Research Group, Department of Evolutionary Zoology University of Debrecen Debrecen Hungary; ^2^ Juhász‐Nagy Pál Doctoral School of Biology and Environmental Sciences University of Debrecen Debrecen Hungary; ^3^ MTA‐DE “Lendület” Evolutionary Phylogenomics Research Group Debrecen Hungary; ^4^ Department of Botany University of Debrecen Debrecen Hungary; ^5^ MTA‐DE Behavioral Ecology Research Group, Department of Evolutionary Zoology University of Debrecen Debrecen Hungary

**Keywords:** clonal reproduction, facultative sexuality, phenotypic plasticity, population genomics, RAD‐seq

## Abstract

Facultative sexual organisms combine sexual and asexual reproduction within a single life cycle, often switching between reproductive modes depending on environmental conditions. These organisms frequently inhabit variable seasonal environments, where favorable periods alternate with unfavorable periods, generating temporally varying selection pressures that strongly influence life history decisions and hence population dynamics. Due to the rapidly accelerating changes in our global environment today, understanding the population dynamics and genetic changes in facultative sexual populations inhabiting seasonal environments is critical to assess and prepare for additional challenges that will affect such ecosystems. In this study, we aimed at obtaining insights into the seasonal population dynamics of the facultative sexual freshwater cnidarian *Hydra oligactis* through a combination of restriction site‐associated sequencing (RAD‐Seq) genotyping and the collection of phenotypic data on the reproductive strategy of field‐collected hydra strains in a standard laboratory environment. We reliably detected 42 MlGs from the 121 collected hydra strains. Most of MLGs (*N* = 35, 83.3%) were detected in only one season. Five MLGs (11.9%) were detected in two seasons, one (2.4%) in three seasons and one (2.4%) in all four seasons. We found no significant genetic change during the 2 years in the study population. Clone lines were detected between seasons and even years, suggesting that clonal lineages can persist for a long time in a natural population. We also found that distinct genotypes differ in sexual reproduction frequency, but these differences did not affect whether genotypes reappeared across samplings. Our study provides key insights into the biology of natural hydra populations, while also contributing to understanding the population biology of facultative sexual species inhabiting freshwater ecosystems.

## Graphical abstract

Facultative sexual organisms frequently inhabit variable seasonal environments, where favourable periods alternate with unfavourable periods, generating temporally varying selection pressures that strongly influence life history decisions and hence population dynamics. In this study we aimed at obtaining insights of the seasonal population dynamics of the facultative sexual freshwater cnidarian *Hydra oligactis* through a combination of restriction site‐associated sequencing (RAD‐Seq) genotyping and the collection of phenotypic data on the reproductive strategy of field‐collected hydra strains. Our findings suggest that facultative sexual organisms are genetically plastic enough to maintain different reproductive strategies (asexual and asexual reproduction) in parallel, which may give them a significant advantage in predictably changing environments, thus increasing their adaptive capacity even in the face of unpredictable changes.

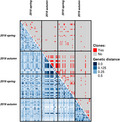

## 1 INTRODUCTION

Facultative sexual organisms, such as Cnidarians or Cladocerans, are very important elements of marine and freshwater ecosystems, and their large numbers make them essential for the construction of aquatic food web system. Facultative sexual organisms often inhabit ephemeral or highly seasonal environments where favorable periods alternate with unfavorable ones in either predictable or unpredictable ways. In favorable periods, clonal reproduction often occurs, allowing the maximal utilization of available resources (Hadany & Otto, 2007; Stelzer, 2012; Stelzer & Lehtonen, 2016). On the contrary, the onset of adverse periods often triggers sexual reproduction, which ultimately results in the formation of resting eggs, such as in case of aphids (Simon et al., 2002), rotifers (Schröder, 2005; Stelzer & Lehtonen, 2016), water fleas (Tessier & Caceres, 2004), and hydras (Steele et al., 2019). The study of such reproductive systems has become more important nowadays, as the frequency of extreme environmental conditions has significantly increased due to recent climate change, which resulted in the large‐scale disappearance or extinction of ecosystems engineered by facultative clonal species (e.g., coral reefs and mangrove forests; Carpenter et al., 2008; Polidoro et al., 2010; Waycott et al., 2009). However, genetic variation of sexually produced clonal lines, coupled with the capacity of asexual reproduction to achieve quick population growth, could enable such species to better adapt to the challenges posed by climate change and sustain high population sizes even under changing conditions (Pistevos et al., 2011).

The population genetic characteristics of facultative sexual organisms differ from those of obligate sexual organisms, partly because increase in their population size is not necessarily accompanied by the emergence of new genotypes and partly because selection affects them in different ways. For facultative sexual organisms, Nunney's “lineage‐selection” model suggests that solely asexual lines may enjoy short‐term benefits (rapid exploitation of resources and high reproduction rates), but suffer disadvantages on the long term due to higher extinction rates compared with obligately sexual lines (Nunney, 1989). This lineage‐selection model is probably even more important in a changing environment (e.g., seasonal habitats), as the creation of resistant formulas (e.g., resting eggs) is linked to sexual reproduction (e.g., in Daphnias, Decaestecker et al., 2009; in rotifers, Stelzer, 2012; or in hydras, Steele et al., 2019) and asexually reproducing lines can be easily removed by natural selection from the population when conditions deteriorate (Stelzer & Lehtonen, 2016). However, genotypes that reproduce only sexually can also be at a significant disadvantage in such an environment (Kokko, 2020), as they cannot reach a sufficiently large number of individuals during the optimal growth period. As a result, it is expected that in these populations genotypes that follow a strategy in which both modes of reproduction appear will prevail. In this case, the differences in the frequency and timing of their sexual reproduction (phenotypic plasticity of reproduction, Stelzer & Lehtonen, 2016), as has already been observed in rotifers (Tarazona et al., 2017), can greatly influence the survival of these genotypes. Thus, a special, seasonally changing genetic structure can emerge, in which the main driving force is the intermittent but large‐scale genetic recombination due to sexual reproduction and clonal selection.

The genetic structure of populations of facultative sexual organisms, such as *Daphnia* species (cyclically parthenogenetic), is determined by the genetic consequences of combining sexual and asexual reproduction in the same life cycle (Carvalho, 1994; De Meester et al., 2006; Decaestecker et al., 2009), the pattern of which can be greatly influenced by the seasonal environment. At the start of the growing period (usually in spring), hatching of sexually produced dormant eggs increases the genetic variation in the population (De Meester, 1996; De Meester et al., 2006). In contrast, asexual reproduction during the favorable season results in erosion of clone diversity through natural selection and extinction of clones, thus ultimately leading to lower genetic variation and deviations from Hardy–Weinberg equilibrium by the end of the favorable period (“clonal erosion,” De Meester, 1996; De Meester et al., 2006; Ortells et al., 2006; Tessier et al., 1992). Directional/stabilizing selection in such natural populations can build up significant genetic imbalances in polygenic traits during the period of asexual reproduction. Levels of genetic disequilibria have a strong effect on the genetic structure of natural populations, so that selection during the period of asexual reproduction can erode the expressed genetic variation in quantitative traits. Thus, when genetic disequilibrium exists, a portion of the total genetic variation is “hidden” by this disequilibrium. Therefore, significant amounts of “hidden” genetic variation may be present in such populations where the individuals are clonally reproducing for long time, but in unfavorable periods undergo sexual reproduction(Deng & Lynch, 1996; Pfrender & Lynch, 2000). Studies to date have shown that a reduction in the disequilibrium in case of a purely additive‐based polygenic trait converts up to 50%–75% of the “hidden” genetic variation into an expressed genetic variation (Lynch & Gabriel, 1983). Moreover, even a single random mating is sufficient to reduce the gametic phase imbalance, thus increasing the expressed genetic variations in the population. Natural selection acts differently during the two reproductive phases (King & Schonfeld, 2001; Pfrender & Lynch, 2000), because during the asexual phase, all genes belong actually to one linked group, and so selection affects the whole genome. For this reason, clonal selection also shapes the interaction of genetic variation, in contrast to sexual reproduction, which interrupts the relationships of these linked alleles (Decaestecker et al., 2009). De Meester et al. (2006) identify three key factors (population size, length of the favorable season, and strength of clonal selection) that influence the genetic structure of cyclical parthenogens. The extent to which “clonal erosion” affects the genetic structure of cyclic parthenogens is primarily determined by the three factors mentioned above (De Meester et al., 2006). The remarkable effect of clonal erosion on seasonal changes in the genetic composition of populations has already been shown by (Yin et al., 2010) in Daphnia, where the survival of clonal genotypes varied from population to population, significantly altering the genotype composition of the following year's population. Life history strategies and physiological condition (e.g., age, body size, nutrition‐state, and stress‐state) of individuals can also significantly influence genetic composition of populations (genotype diversity), as these are factors that clearly play a role in initiating sexual reproduction and thereby alter population dynamics (Hadany & Otto, 2007). However, the exact role of genotype‐specific life history strategies has not been adequately explored in previous studies.

*Hydra oligactis* living in seasonal habitats of the temperate zone can serve as an excellent model animal for the ecological study of the reproductive system of facultative sexual organisms. *H. oligactis* polyps reproduce asexually (budding) throughout much of the year but switch to sexual reproduction in response to cooling (Reisa, 1973). In natural habitats, within the distribution range of *H. oligactis,* sexual reproduction occurs from late summer to December (Ribi et al., 1985; Sebestyén et al., 2018; Welch & Loomis, 1924). During sexual reproduction, persistent/diapausing embryos are produced which can tolerate desiccation and freezing (Steele et al., 2019). Based on our observations, however, in some of the natural populations, asexually and sexually reproducing individuals occur simultaneously before the unfavorable periods and adults can survive the unfavorable periods in large numbers (Miklós et al., 2021; MM & JT, personal observations). However, the contribution of sexual individuals with diapausing eggs to the genetic structure and seasonal population dynamics of this species has not been studied so far.

In a recent study (Miklós et al., 2021), we described genetic structure underlying sexual and asexual reproductive strategies in *H. oligactis* in a spatial setting. That study showed substantial phenotypic plasticity in the mode of reproduction, with coexisting hydra polyps belonging to the same genotype often differing in their mode of reproduction. However, it is still unclear whether more subtle genotypic variance in propensity of reproduction exists, and how this variation affects population dynamics. This is because field studies are less likely to detect small differences in life history among clonal lines due to: I) difficulties in designing an adequate sampling strategy and the small number of individuals per clonal line that are often obtained from random sampling of natural populations (Halkett et al., 2005) and II) the fact that individuals in their natural environments can be exposed to a diversity of environmental effects, generating variation within clonal lineages that masks potential genotypic variation (Deng & Lynch, 1996; Thorson et al., 2017). To solve these problems, studying a large number of clonally descended individuals from multiple genotypes kept under standard laboratory conditions would be required. In this study, we sought to fill this gap by simultaneously identifying the temporal genetic variation in a *H. oligactis* population and the reproductive strategies of the genotypes involved. To this end, we used data on reproductive strategy from laboratory strains that were collected from a single population during spring and autumn in two years (four collections in total) and genotyped these strains using restriction site‐associated DNA sequencing (RAD‐Seq). The population we sampled is a small, shallow temperate lake where a *H. oligactis* population persists year‐round (although with a highly variable population size; MM & JT, pers. obs.). Asexual individuals can be observed year‐round (with peak population sizes during late winter/early spring), while sexual individuals are generally detected between late October and early December (Sebestyén et al., 2018). However, sexual and asexual individuals coexist during the autumn sexual period and we do not know how these contribute to the changes in the genetic composition of the population. We used the combination of laboratory‐collected phenotype data and RAD‐Seq genotyping to ask first if clone lineages survive the unfavorable period and how this affects the genetic composition of a population in such a seasonal environment in temperate climate. Second, we wanted to explore the role of different genotypes in the propensity for sexual reproduction and its timing, as well as in the associated population dynamics.

## 2 MATERIALS AND METHODS

### 2.1 Study design and field collection of hydra polyps

**FIGURE 1:**
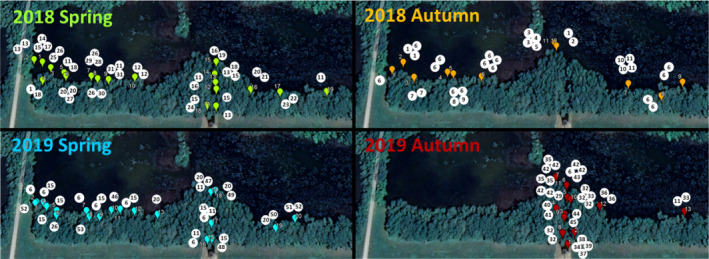
Map showing the collection point of *Hydra oligactis* polyps in two distinct seasons (spring vs. autumn) in two consecutive years (2018 and 2019, four samplings in total) from a single population in Central Hungary. The numbers in the white circles represent the identified MLGs (genotypes) from that sampling point

Laboratory *Hydra* strains were established from a single oxbow lake in Eastern Hungary (Tiszadorogma, 47.6712 N, 20.8641 E) on four dates: May 31, 2018 (henceforth called Spring 2018), October 1, 2018 (Autumn 2018), May 16, 2019 (Spring 2019), and September 24, 2019 (Autumn 2019). This oxbow lake, about 15 hectares in size, is a shallow (average 1 meter deep) eutrophic water body that is directly connected to the Tisza River through a canal. The water temperature in the lake can rise above 25 ° C in the warmest months (even though the lake is surrounded by woody vegetation, which provides substantial shade), while it stays below 12°C between October and April. The data on laboratory strains used here were collected as part of a previous study aimed at comparing spring‐ and autumn‐collected hydra strains (Tökölyi et al., 2021). Briefly, we collected polyps from multiple locations along the shoreline (distance between locations at least 2 meters), with the following sampling intensity: spring sampling in 2018 from 19 points; autumn sampling in 2018 from 11 points; spring sampling in 2019 from 20 points; autumn sampling in 2019 sampling from 13 points (these are shown in Figure 1). We recorded the GPS coordinates of each collection point (Garmin GPSMAP 60 CSx), (Figure 1; Table S1). *Hydra* polyps were collected from free‐floating and submerged macrophytes (most often *Ceratophyllum demersum*, *Ceratophyllum submersum*, *Myriophyllum spicatum, and Stratiotes aloides*), then were put in a Falcon tube with lake water. On the day of collection, animals in Falcon tubes were transported to the laboratory in a cool box, where they were identified by stereomicroscopy (with Euromex StereoBlue stereo microscope) based on morphology—tentacle length / body length, the presence of stalk, and tentacle formation in buds (Schuchert, 2010).

### 2.2 Laboratory maintenance of hydra strains and sex induction

Hydra polyps transported to the laboratory were immediately moved to hydra medium (M‐solution: 1 mM Tris, 1 mM NaCl, 1 mM CaCl_2_, 0.1 mM KCl, and 0.1 mM MgSO_4_ at pH 7.6; Lenhoff, 1983). We selected up to five polyps from each location/collection to create strains from them through their budding (asexual reproduction). Both natural‐collected polyps and their asexual offspring were kept individually in 6‐well plastic plates, which contained 5 ml M‐solution per well. Experimental animals were asexually propagated for 10 weeks in the first phase and then placed into cold circumstances to induce sexual reproduction in the second phase. Details of the standard living conditions of the hydra polyps, both for asexual reproduction phase and cooling phase, can be found in Tökölyi et al. (2021). To keep samples at a manageable size, the maximum number of polyps/strain was set up to *N* = 18 were retained to collect data on reproductive mode. Experimental animals were kept for 5 months under second phase and were checked twice per week under a stereo microscope (with Euromex StereoBlue stereo microscope) to detect the start of gonadogenesis.

### 2.3 Drying and DNA extraction

Asexual buds detached from experimental animals were used to genotype strains. They were dried using silica gel and stored at room temperature to preserve DNA quality (see Miklós et al., 2021). Genomic DNA from *H. oligactis* individuals was isolated using a standard mammalian nucleic acid extraction protocol (detailed description of used method, see in supplementary of (Miklós et al., 2021); [Supplementary Methods 1]). The samples were stored in a freezer (−20°C) until libraries were prepared.

### 2.4 RAD‐Seq library preparation

Details of the library preparation protocol can be found in the supplement of (Miklós et al., 2021); (Supplementary Methods 1). Quality and quantity of the library were checked with Bioanalyzer (High‐Sensitivity DNA Kit). Libraries were sequenced on an Illumina NovaSeq platform (paired‐end, 150 nt) at NovoGene (Beijing, China). Our samples were sequenced in three separate RAD libraries (56 strains in the first, 53 strains in the second, and 30 strains in the third). In all library preparations, we used the same methodologies, but in the third library, only 15 cycles were used during PCR amplification, to reduce the presence of PCR duplicates (the number of PCR cycles used to create the first two libraries was 18). Finally, GenBank association numbers associated with identified specific genotypes were recorded (Table S1).

### 2.5 Sequence processing and decontamination

First, raw Illumina reads were processed using Stacks *process_radtags* pipeline (Catchen et al., 2013; Miklós et al., 2021). We first ran the pipeline with default parameters and calculated the GC content of the resulting RAD loci with the BBmap suite of tools (https://sourceforge.net/projects/bbmap/). This showed a secondary GC peak suggesting sequence contamination from bacterial DNA. Therefore, we performed in silico decontamination using the NCBI Basic Alignment Search Tool (BLAST, v. 2.7.1; Altschul et al., 1990) to map sequences to the NCBI nucleotide collection database (*nt*, downloaded 31st March 2021), with *blastn* task and E‐value cutoff set to 1e‐05. RAD loci whose best match was a cnidarian sequence in the *nt* database or showed no hit were retained, while loci that mapped to any other taxonomic group were separated to form a contaminants database. We then mapped our paired‐end demultiplexed reads to this contaminants database with Bowtie 2 *‐‐very sensitive* and retained only unmapped reads. Sequence handling was carried out with the BBmap suite of tools (https://sourceforge.net/projects/bbmap/), while taxonomic annotation was carried out with the *taxonomizr* R package (v. 0.5.3; Sherrill‐Mix, 2019); R Core Team, 2020).

Next, we ran the de novo pipeline on the unmapped reads, setting the parameters minimum depth of coverage required to create a stack (−m), the number of mismatches allowed between stacks within individuals (−M), and the number of mismatches allowed between stacks between individuals (−n). The optimal value for these parameters depends on sequencing error, genetic polymorphism and level of ploidy, among others (Paris et al., 2017). We set all three parameters (−*m*, −*M*, and ‐*n*) to based on our previous study (Miklós et al., 2021) that included some individuals from the population now being studied and included a detailed exploration of the parameter space in this study system.

From the resulting set of loci, we retained those that were shared by at least 80% of the samples. Additional filtering was performed on the resulting locus catalog using VCFtools (Danecek et al., 2011), with the following parameters: We required a minor allele count (−‐mac) of 3, minor allele frequency (−‐maf) of 0.05, minimum genotype quality (−‐minGQ) of 30, minimum read depth (−‐minDP) of 10 and mean depth values across individuals (−‐max‐meanDP) less than 97.5% of the mean depth values for the whole dataset. Then, we checked individual missingness, removed samples with >50% missingness and repeated all filtering steps without these samples, following Cerca et al. (2021).

### 2.6 Clone detection and sibship reconstruction

To identify clones, we first inspected the spectrum of genetic diversity, that is, the distribution of pairwise genetic distances of the samples (Rozenfeld et al., 2007). Clonally derived individuals in theory should be genetically identical to each other. However, due to sequencing errors and somatic mutations, they frequently show a distribution of genetic distances >0, but less than the genetic distance of distinct genotypes, which often results in a bimodal distribution (Figure S1). We also used the software COLONY (v. 2.0.6.6; Jones & Wang, 2010) to infer clones more formally using an optimized threshold that takes into account mistyping rates, missing data, and the number and allele frequencies of markers (Wang, 2016). The method implemented in COLONY uses a likelihood framework to assign individuals to candidate relationships of clone mates and close competitive relationships (e.g., full sibships) and has been shown to accurately identify individuals belonging to the same multilocus genotypes (MLGs) through simulations (Wang, 2016). All individuals were included as potential offspring in the analysis, as the presence of clonality in *Hydra* implies that generations can be overlapping and there is no unequivocal way to assign candidate parents. These potential offspring were then assigned into clonal lineages. For the COLONY analysis, we used a full‐likelihood‐pair‐likelihood score combined (FPLS) method, assumed a polygamous mating system for both parents and kept all other parameters at their default values. Initial error rates were set to 0.01 for both allelic dropout rate and other error rate of each locus in COLONY.

### 2.7 Seasonal genetic structure

To visualize the seasonal distribution of sample genotypes, minimum spanning networks (MSN) were constructed using the function *poppr.msn* (Kamvar et al., 2014) in R. The network was constructed on the basis of genetic distance matrix calculated in *ape*'s R package *dist.gene* function, with pairwise deletion of missing loci. These relationships were visualized with MSN (because for clonal organizations it can be a better visualization tool than tree drawing methods) generated using the R packages *igraph* and *poppr* (Csardi & Nepusz, 2005; Kamvar et al., 2014).

Basic population genetic statistics (expected heterozygosity, observed heterozygosity, fixation index, allelic richness, the number of private alleles and clonal richness, evenness, and diversity) were calculated for two datasets. In the first analysis, all samples were included. However, as *H. oligactis* is a clonal species, the presence of clones can bias the results of population genetics statistics. Therefore, we also prepared a reduced dataset that included one strain from each MLG per sampling (based on results obtained from COLONY) and repeated the calculations.

Expected heterozygosity, observed heterozygosity, and fixation index were calculated in the *hierfstat* R package (v. 0.5–7; Goudet et al., 2020), allelic richness was calculated with the *PopGenReport* R package (v. 3.0.4; Adamack & Gruber, 2014), while the number of private alleles were obtained with the *poppr* R package (v. 2.9.0; Kamvar et al., 2014). Clonal richness was calculated as R = (G‐1) / (N‐1), where G is the number of MLGs and N is the number of strains (Dorken & Eckert, 2001). Evenness and Shannon–Wiener diversity were calculated in *poppr*.

To detect genetic structure with respect to sampling dates, we performed discriminant analysis of principal components (DAPC; Jombart et al., 2010) on the reduced dataset. DAPC analysis was performed using the *adegenet* (v. 2.1.1) package in R (Jombart, 2008; R Core Team, 2020). The number of principal components used in the DAPC analysis was set to 13 following alpha‐score optimization and we generated inertia ellipses encompassing ~67% of the cloud of points for each population. For the DAPC analysis, we only included 1 individual from each MLG. As DAPC might be sensitive to missing data, we repeated this analysis on a dataset that resulted from a more stringent selection of loci (we included only loci that were shared across 90% of the samples).

Finally, we performed analysis of molecular variance (AMOVA) between to see whether there is significant genetic structure between seasons and samplings. In the AMOVA analysis, sampling (i.e., 2018 spring, 2018 autumn, 2019 spring, or 2019 autumn) was nested within season (spring or autumn), and we calculated the variance components associated with these factors (σ^2^), as well as p‐values and the proportion of variance explained by the factors (Φ‐statistics, corresponding to fixation index, F_st_). AMOVA was implemented in *R's pegas* (v. 0.10) package (Paradis, 2010) using 1000 permutations.

### 2.8 Genetic structure and reproductive mode

To find out whether genotypes differ in sexual propensity, we fitted a generalized linear mixed model (GLMM) with binomial distribution to the phenotype data collected from the polyps. In the model, mode of reproduction was dependent variable, season and polyp age were explanatory variables and genotype (as inferred from the Colony analysis) was included as a random factor. Then, we used the *get_variance* function from the R package *insight* (Lüdecke et al., 2019) to extract variance components associated with the fixed and random effects, as well as the residual variance. From this, we calculated the proportion of variance explained by the random factor (MLG ID), to find out the degree to which genotype identity contributes to variation in sexual propensity.

## 3 RESULTS

### 3.1 Establishment of field‐collected strains in laboratory

We established strains from *N* = 211 polyps (*N* = 54, 40, 59, 58, respectively, for the four collection dates). However, some of these strains were lost due to mortality before yielding usable data and DNA samples. A total of 138 strains were genotyped (*N* = 40, 30, 38, 30 for the four sampling occasions).

### 3.2 Read statistics and decontamination

There were altogether 699.8 million raw paired‐end reads. From these raw reads, 93.6% were retained after filtering for low‐quality reads, adapter contamination, ambiguous barcodes, and ambiguous RAD‐tags. There were an average of 4.7 million reads per sample (range 2.3–20.9 million).

Running the Stacks de novo pipeline with default settings identified 1,602,803 million RAD loci. 46.9% percent of these loci showed no hit in the *nt* database, a further 13.6% percent mapped to cnidarian sequences. The remainder mapped to other taxonomic groups and were filtered out to form a contaminants database. The top contaminants were Pseudomonadales and Burkholderiales (Figure S1), two bacterial orders that are commonly found within the *Hydra* microbiome (Fraune et al., 2015). After removing presumed contaminant loci, the secondary GC peak diminished substantially (Figure S1).

The proportion of read pairs identified to be PCR duplicates after removal of contaminants was 27.2%. Mean coverage after removing contaminants and PCR duplicates was 13.7x per sample (range: 4.1x–25.7x).

Running Stacks de novo with these final settings, we obtained 1417 RAD loci after filtering. Six samples (all of them from the 2019 spring collection) had individual missingness >50% and were removed. Repeating the filtering of loci without this sample resulted in a final set of 2257 loci. Individual missingness was 15.2% (range: 2.3–54.1%), with 90% of samples having a missingness <26.5%.

### 3.3 Clone detection

Inspection of the spectrum of genetic diversity showed no clear threshold to delineate multilocus genotypes (Figure S2). While there was a clear peak of low genetic distance (less than ~0.06), we also detected a smaller, secondary peak at genetic distance ~0.11. This secondary peak could stem from the fact that genotyping error rates are higher in some of the samples (as seen in our previous study for samples with low coverage; Miklós et al., 2021), or because somatic mutations are more prevalent in some of the samples. Given that we used very stringent filtering of SNPs (minimum genotype quality of 30 and minimum read depth of 10), we think that genotyping error rates in general should be low. Nonetheless, the presence of this secondary peak in the spectrum of genetic diversity makes the identification of clones more difficult.

**FIGURE 2:**
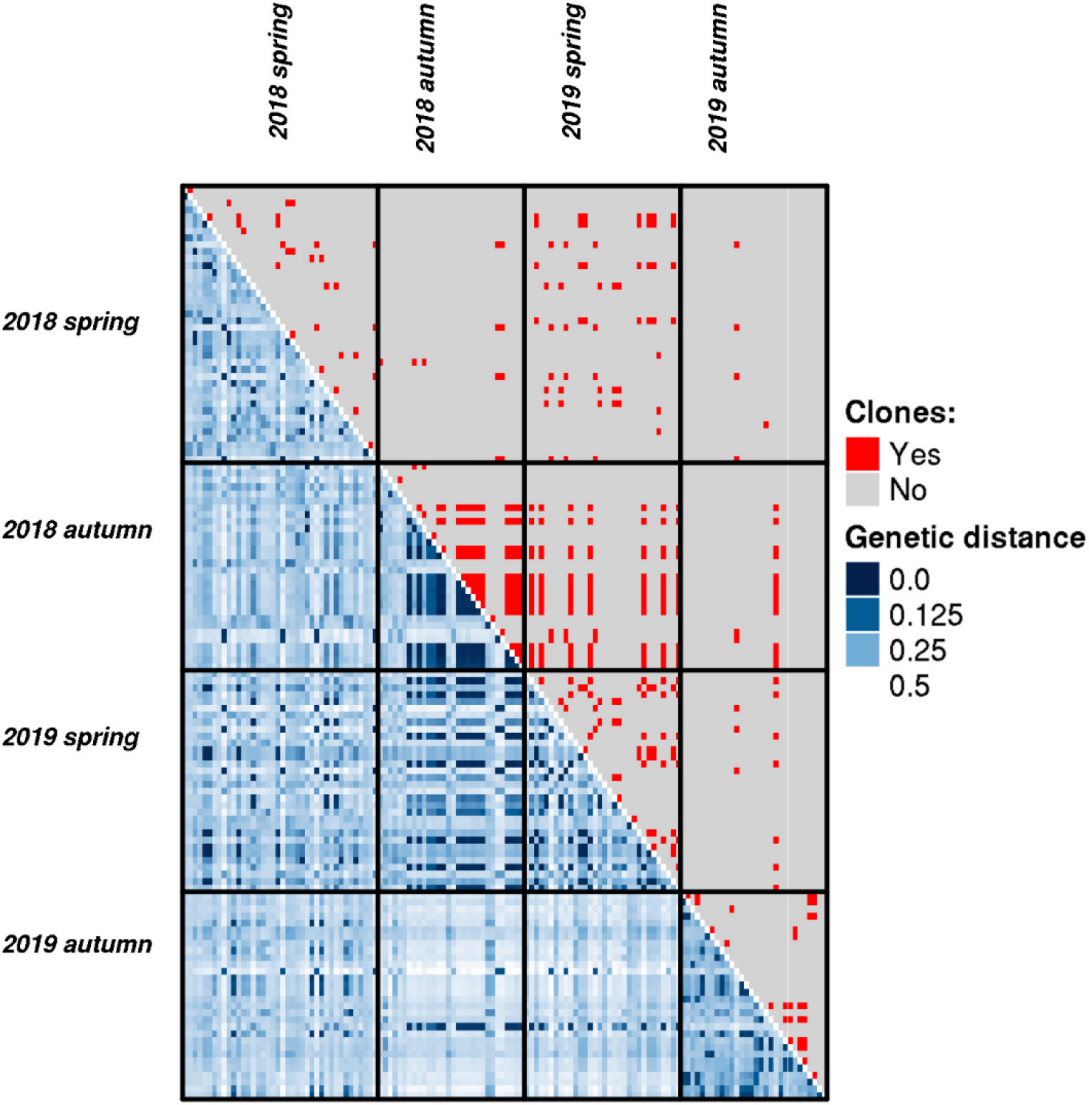
Heatmap showing genetic distance matrix between *N* = 121 genotyped *Hydra oligactis* strains (comparing the genetic distance of two individuals in each small cube in the figure) collected at four distinct time points (lower diagonal). Pairs of strains that were inferred to be clones in the COLONY analysis are shown in red in the upper diagonal of heatmap

The COLONY analysis also revealed these difficulties. We identified *N* = 53 multilocus genotypes in the set of *N* = 132 strains included in the analysis. However, 11 of these 53 MlGs (each of them consisting of a single strain) were inferred with a probability <0.9 (while all other MLGs were inferred with probability 1.0). Therefore, we decided to remove these individuals from subsequent analyses as we cannot unequivocally assign them to MLGs. As a consequence, subsequent analyses are presented for *N* = 121 strains belonging to 42 MlGs (Figure 2). Most of these MLGs (*N* = 35, 83.3%) were detected in only one season. Five MLGs (11.9%) were detected in two seasons (MLG 1: 2018 spring and 2018 autumn; MLG 15: 2018 spring and 2019 spring; MLG 20: 2018 spring and 2019 spring; MLG 26: 2018 spring and 2019 spring; MLG 29: 2018 spring and 2019 autumn), one (2.4%) in three seasons (MLG 6: 2018 spring, 2018 autumn and 2019 autumn) and one (2.4%) in all four seasons (MLG 11).

### 3.4 Seasonal genetic structure

Clonal richness and evenness did not show clear seasonal trends, although Shannon–Wiener diversity was somewhat higher in the spring samples. Observed heterozygosity, expected heterozygosity, F_is_, allelic richness and the number of private alleles did also not show marked seasonal trends (Table 1.).

**TABLE 1:** Basic population genetic statistics from 2557 SNPs obtained with RAD‐seq for *N* = 121 *Hydra oligactis* strains established from polyps collected in two distinct seasons (spring vs. autumn) in two consecutive years (2018 and 2019, four samplings in total) from a single population in Central Hungary

					Full dataset (*N* = 121)							Clone‐corrected dataset (*N* = 54)				
Year	Season	N	MLGs	Clonal richness	Clonal evenness	Shannon‐Weiner diversity	H_obs_	H_exp_	F_is_	Allelic richness	Private alleles	H_obs_	H_exp_	F_is_	Allelic richness	Private alleles
2018	Spring	39	21	0.53	0.85	2.88	0.31	0.24	−0.26	1.78	1	0.30	0.23	−0.24	1.44	5
2018	Autumn	30	11	0.34	0.54	1.87	0.34	0.24	−0.41	1.71	0	0.35	0.26	−0.31	1.48	1
2019	Spring	31	12	0.37	0.76	2.14	0.33	0.24	−0.36	1.74	0	0.34	0.25	−0.29	1.47	0
2019	Autumn	21	8	0.35	0.82	1.86	0.38	0.26	−0.41	1.75	8	0.37	0.26	−0.34	1.50	9

**FIGURE 3:**
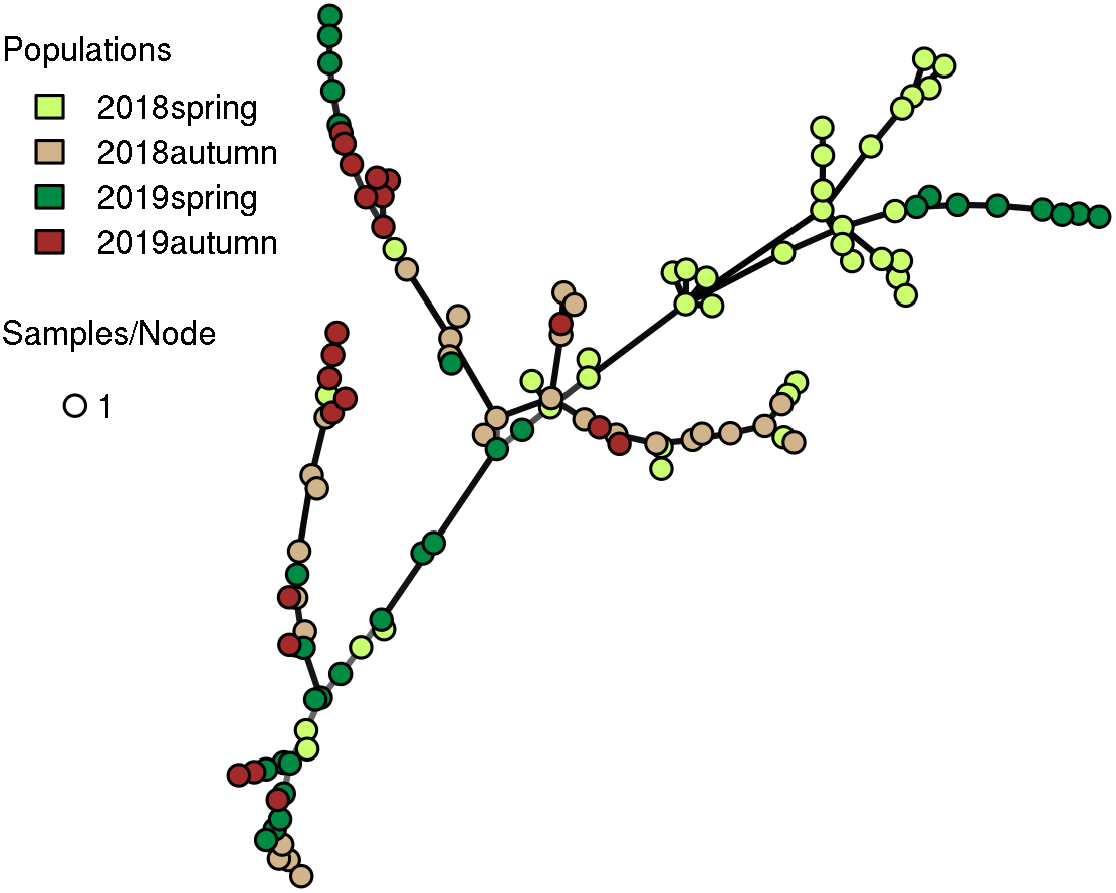
Minimum spanning network based on a dissimilarity matrix as calculated in *poppr* of *N* = 121 *Hydra oligactis* strains established from polyps collected in two distinct seasons (spring vs. autumn) in two consecutive years (2018 and 2019, four samplings in total) from a single population in eastern Hungary. Node colors represent sampling occasions. Edges length is arbitrary

The minimum spanning network showed that individuals collected from different seasons did not show significant separation from each other, but clustered into 4 major branches (Figure 3.). Of these, three branches contained individuals from all collections, while in the fourth branch we found only polyps from spring collections (spring 2018 and spring 2019).

**FIGURE 4:**
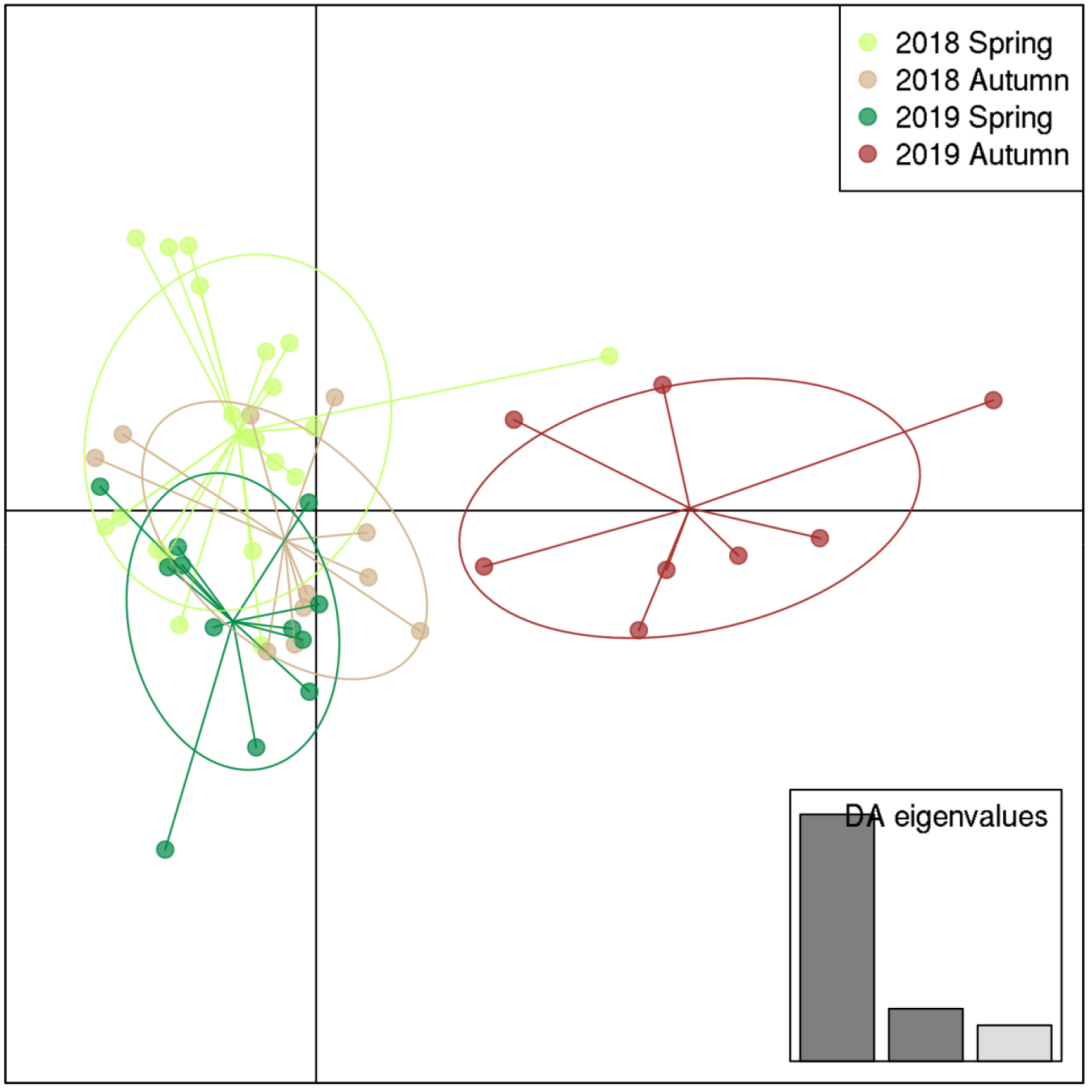
Differentiation of *Hydra oligactis* strains derived from four distinct sampling types based on discriminant analysis of principal components (DAPC) performed on a reduced dataset containing one individual from each MLG per sampling. The DAPC was constructed using 13 principal components (PCs). The inset shows eigenvalues for the discriminant analysis

The DAPC analysis likewise indicated substantial overlap between seasons, with the last sample being more distinct from the rest (Figure 4). We repeated this analysis on a more stringent selection of loci (those shared across 90% of the samples; 269 RAD loci with average missingness of 8%, range: 1%–43%) and obtained very similar results (Figure S3).

Reflecting the DAPC results, we found no significant genetic structure among seasons (σ^2^ = −0.002, *p* = .648, Φ = −0.03), but a significant genetic structure between samplings (σ^2^ = 0.005, *p* = .004, Φ = 0.056) based on AMOVA.

### 3.5 Genetic structure and reproductive mode

Reproductive mode was inferred based on data from *N* = 921 polyps (that belonged to the 121 genotyped strains). There were on average 7.74 polyps per strain to estimate reproductive mode (range: 1–18). We could not estimate reproductive mode for two strains where all polyps were lost before data collection. After genotyping, there were 21.9 polyps per MLG to estimate reproductive mode (range: 2–136).

**FIGURE 5:**
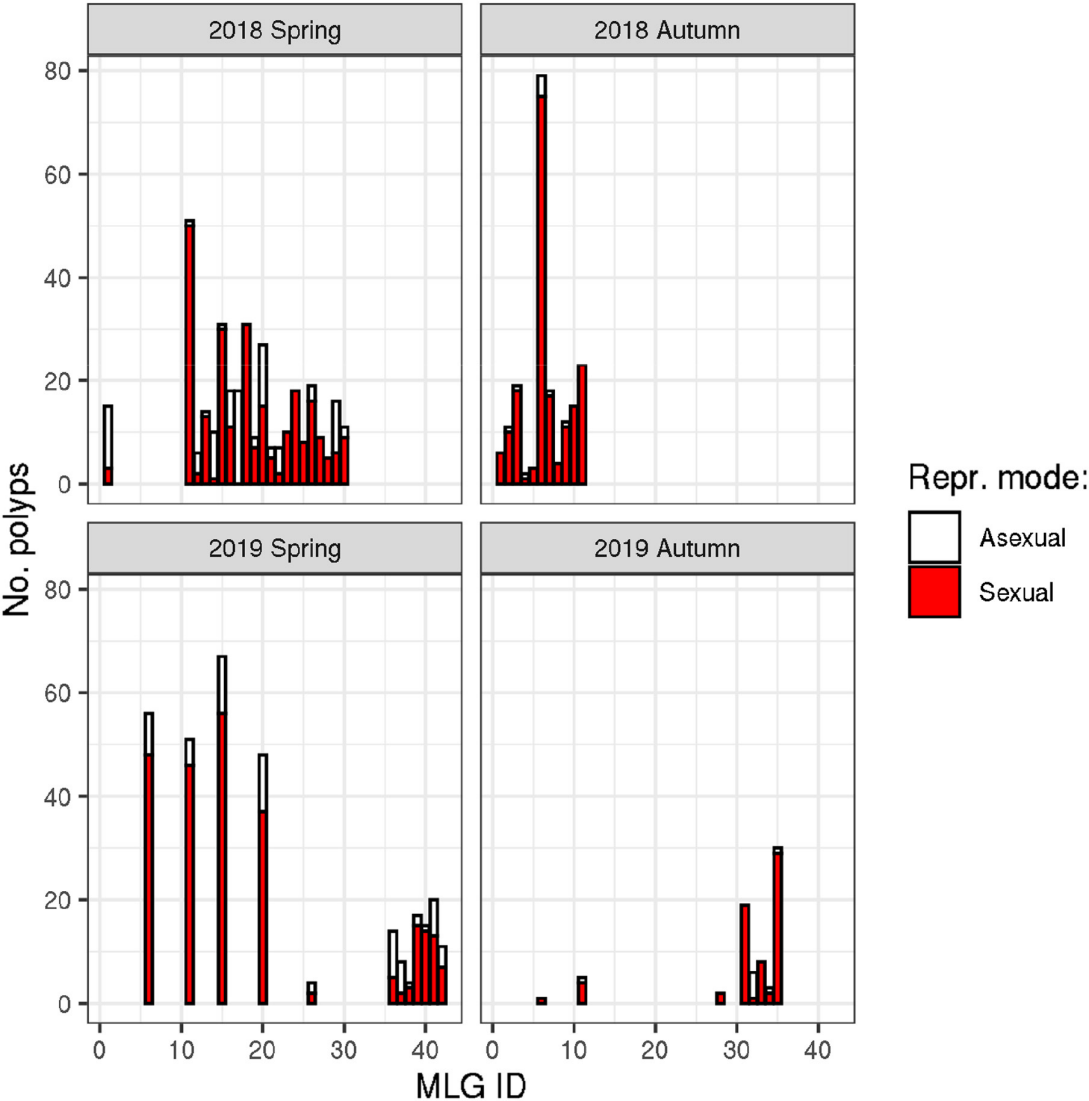
Number of sexual and asexual *H. oligactis* individuals in strains established from polyps collected at four consecutive time points (2018 spring, 2018 autumn, 2019 spring, and 2019 autumn) and tested under standard laboratory conditions. Each par represents a different MLG, as inferred by RAD‐seq genotyping

A single MLG (2.4%) contained only asexual polyps 11 out of 42 MlGs (26.2%) contained only sexual individuals, and the other 30 MlGs (71.4%) contained a mixture of sexual and asexual individuals (Figure 5.). The average proportion of sexual individuals per MLG was 74.0%.. The proportion of variance in reproductive mode explained by MLG ID based on the binomial GLMM was 46.7%, while season and polyp age explained 10.1% of variance in total.

The proportion of sexual individuals in MLGs that were found in multiple samplings was 0.81 ± 0.20 (mean ± SD, *N* = 7), compared to 0.73 ± 0.31 in MLGs that were detected in a single sampling (*N* = 35); however, this difference was statistically nonsignificant (Kruskal test, χ^2^ = 0.014, *p* = .905).

## 4 DISCUSSION

The primary objective of our study was to investigate how the genetic composition of a population of a common freshwater facultative sexual organism, *Hydra oligactis*, changes in a temperate seasonal environment. We detected (1) limited changes in the seasonal population genetic composition and found that: (2) some hydra clone lines can survive between years and seasons. Furthermore, we also found that (3) distinct genotypes differ in sexual reproduction frequency. Finally, (4) the above differences did not affect whether these genotypes reappear between samplings. We discuss the consequences and circumstances of these findings below.

We found no clear evidence for an abundance of spring genotypes in the hydra population, despite the fact that it would be a logical consequence of their high rate of sexual reproduction in winter (generation of new genotypes) and has been described previously for other facultative sexual organisms (Daphnia; De Meester, 1996; De Meester et al., 2006). Based on previous studies, we would have expected that asexual reproduction during the favorable period would lead to a reduction in clonal diversity (through natural selection and random extinction of clones), resulting in lower genetic variation at the end of the favorable period and greater deviations from Hardy–Weinberg equilibrium (clonal erosion; De Meester, 1996; De Meester et al., 2006; Tessier et al., 1992), but in our study hydra population, we were not able to detect this clonal erosion clearly. Several factors could be behind this observation. One possibility is that the contribution of sexually produced offspring is low compared with asexually produced ones and there is no marked increase in the abundance of new genotypes during spring. Another possible explanation for the lack of clonal erosion is that no strong selection factors appear in the study population to induce clone extinction. For example, oversupply of food organisms available throughout the favorable period (Cladocerans and Copepods) could generate a relatively stable environment where most clonal lines can survive, thus eliminating clonal erosion. However, it is also possible that our sample size was too low to detect clonal erosion. This could be especially the case if only a few persistent eggs hatch in the spring and grow in a large asexual population, or clonal selection is very week (i.e., the signal for clonal erosion is weak), in which case we would need a very large sample size to detect it. Unfortunately, we do not know much about the reproductive biology of the species in natural populations (neither the rate of eggs hatch in the spring, nor how long they are viable, etc.). However, our data are more in line with a scenario of weak clonal erosion in this system, as we did not observe a clear decrease in the diversity of clones as a function of season, but we did find MLGs that clearly survive the winter. Finally, another reason could be that we could not actually identify all clones when analyzing the data. Identifying clones in this study proved difficult because of the relatively high frequency of pairs of individuals that had a genetic distance intermediate between clones and distinct genotypes. We used very stringent filtering of SNPs to increase genotyping accuracy, which further reduced sample size. However, all retained strains were assigned to MLGs with a high certainty in the COLONY analysis; therefore, we think that the obtained results reflect true biological patterns. In general, we can conclude that there is definitely a set of genotypes in the population that persists for a relatively long time regardless of seasonality, without significant restructuring of the genetic composition with seasons, as we did not find significant genetic variation among spring and autumn‐collected samples. In addition, we observed a significant (although not large) change in genotype composition in the second autumn, but the exact reason for this is not known (it is possible that this was a consequence of an unusually warm summer before, which may have enhanced and modified the usual selection effects).

We reliably demonstrated that some clonal lines can survive seasons and even years, contrary to the literature that assumes that *H. oligactis* polyps die in winter due to freezing waters, and only survives in sexual enduring forms (Brien, 1953). The survival strategy based on persistent eggs produced after sexual reproduction is common in aquatic invertebrate species that can reproduce facultatively in temperate or cold climates, such as Rotifers (Walsh et al., 2014) or Cladocerans (Decaestecker et al., 2009). In climates where mild winters often occur, the survival of clones can also have a significant adaptive advantage in terms of a given clone genotype, resulting in a better return on investment in winter asexual reproduction. It has already been observed in Cladocerans that greater investment in asexual reproduction may be adaptive in a relatively mild winter climate where the risk of freezing is small and the adaptive value of dormancy may be low (Tessier & Caceres, 2004). The situation may be similar for other temperate species, such as hydras. From a population dynamics point of view, such surviving individuals can significantly influence the composition of the early spring population, as they may even begin their clonal reproduction while the other genetic lines are still waiting in resting form. Thus, such hydra lines may start with a larger number of individuals over the newly emerging genotypes, thus gaining a significant advantage in resource exploitation. Nonetheless, our dataset collected over 2 years suggests that this survival of clonal lines may not be high enough to entirely displace genotypes with a higher sexual propensity.

We also observed that different genotypes differ in sexual reproduction frequency. Based on previous studies (Tökölyi et al., 2017; Tomczyk et al., 2015), genotypic differences may contribute to variation in the propensity of sexual reproduction in hydras because, under normal laboratory conditions, *H. oligactis* strains express differences in the probability of initiating sexual reproduction and in post‐sexual survival rates (Ngo et al., 2021). Conversely, in another previous study analyzing reproductive mode under field condition, we detected a high rate of phenotypic plasticity in reproduction modes in this species (Miklós et al., 2021). Now, however, it has also been shown that there is a high degree of plasticity in the expression of the mode of reproduction between the different genotypes. Different genotypes show different propensity to initiate sexual reproduction in the same environmental conditions. Thus, due to the fact that individuals in the same genotypes respond differently to the same environmental stimulus (which may even be due to the different physiological status of the individuals; Hadany & Otto, 2007), it may create diverse reproductive strategies in populations (Tökölyi et al., 2021). Therefore, in fact, the observed diversity in reproductive patterns results from a combination of genetic diversity and phenotypic plasticity, which may result in a better adaptability of the population to the changing environment. This may play a significant role in the long‐term change in the genetic composition of such populations, as the genotypes best adapted to a given environmental condition can be the most widespread in a given habitat. Moreover, such a facultative sexual reproductive system is likely to allow rapid adaptation to local conditions through selection of genotypes with an adaptive combination of phenotypic plasticity responses (De Meester et al., 2004). Some studies have already provided evidence for effectual tracking of environmental changes over time in natural Daphnia populations (Cousyn et al., 2001; Hairston et al., 2001). In addition, in previous cases, relevant differences between clone lines (genetic individuals) in some marine species in their response to environmental stimuli, including the choice of mode of reproduction, have been described (Langer et al., 2009; Pistevos et al., 2011). This trait is of great importance for these species in adapting to the environment driven only by selection. This is also important because such metazoan will be more easily able to adapt to rapid global change through natural selection affecting existing genotypic variations (Balanyá et al., 2006; Bradshaw & Holzapfel, 2001), despite the slow onset of their mutational changes (Hoffmann et al., 2003).

Interestingly, differences in genotype traits did not affect the reappearance of specific genotypes in different samples. The simplest explanation for this may be that such a sampling time interval is not sufficient to accurately describe such consequences of population dynamic effects, because random effects may still obscure them. An alternative explanation could be that each genotype is so plastic that even if a significant proportion of individuals in genetic lineages are likely to reproduce sexually, they may also be survived by asexually reproducing polyps, which thus maintain the genetic lineage (bet‐hedging; Simons, 2009; Steele et al., 2019).

## 5 CONCLUSION AND PERSPECTIVE

In conclusion, the above findings suggest that the facultative sexual *H. oligactis* could maintain different reproductive strategies (asexual and asexual reproduction) in parallel, which may give them a significant advantage in predictably changing environments, thus increasing their adaptive capacity even in the face of unpredictable changes. This makes the study of this ability even more relevant today, as ecosystems with populations with these traits could be the key to mitigating ecological damage caused by climate change.

## AUTHOR CONTRIBUTIONS

**Máté Miklós:** Conceptualization (lead); data curation (equal); investigation (lead); methodology (lead); project administration (lead); visualization (supporting); writing – original draft (lead); writing – review and editing (lead). **Levente Laczkó:** Conceptualization (supporting); formal analysis (supporting); investigation (equal); methodology (equal); writing – original draft (supporting); writing – review and editing (supporting). **Gábor Sramkó:** Conceptualization (supporting); methodology (supporting); resources (equal); validation (supporting); writing – original draft (supporting); writing – review and editing (supporting). **Zoltán Barta:** Conceptualization (equal); funding acquisition (equal); methodology (supporting); resources (equal); supervision (lead); validation (equal); writing – original draft (supporting); writing – review and editing (supporting). **Jácint Tökölyi:** Conceptualization (equal); data curation (lead); formal analysis (lead); funding acquisition (lead); investigation (supporting); methodology (supporting); visualization (lead); writing – original draft (equal); writing – review and editing (supporting).

## CONFLICT OF INTEREST

All authors declare that there is no conflict of interest.

## Supporting information


Appendix S1

## Data Availability

Raw DNA sequence reads have been deposited in the US National Center for Biotechnology Information (NCBI) Sequence Read Archive (Accession number PRJNA542036; https://www.ncbi.nlm.nih.gov//bioproject/PRJNA542036). Phenotype data are available in Figshare (https://doi.org/10.6084/m9.figshare.12727673.v1). The R‐scripts of our analysis was uploaded to Github (https://github.com/jtokolyi/Hydra_oligactis_PopDynamics).

## References

[ece39096-bib-0001] Adamack, A. T. , & Gruber, B. (2014). PopGenReport: Simplifying basic population genetic analyses in R. Methods in Ecology and Evolution, 5(4), 384–387. 10.1111/2041-210X.12158

[ece39096-bib-0002] Altschul, S. F. , Gish, W. , Miller, W. , Myers, E. W. , & Lipman, D. J. (1990). Basic local alignment search tool. Journal of Molecular Biology, 215(3), 403–410. 10.1016/S0022-2836(05)80360-2 2231712

[ece39096-bib-0003] Balanyá, J. , Oller, J. M. , Huey, R. B. , Gilchrist, G. W. , & Serra, L. (2006). Global genetic change tracks global climate warming in Drosophila subobscura. Science (New York, N.Y.), 313(5794), 1773–1775. 10.1126/science.1131002 16946033

[ece39096-bib-0004] Bradshaw, W. E. , & Holzapfel, C. M. (2001). Genetic shift in photoperiodic response correlated with global warming. Proceedings of the National Academy of Sciences of the United States of America, 98(25), 14509–14511. 10.1073/pnas.241391498 11698659 PMC64712

[ece39096-bib-0005] Brien, P. (1953). La Perennite somatique. Biological Reviews, 28(3), 308–349. 10.1111/j.1469-185X.1953.tb01381.x

[ece39096-bib-0006] Carpenter, K. E. , Abrar, M. , Aeby, G. , Aronson, R. B. , Banks, S. , Bruckner, A. , Chiriboga, A. , Cortés, J. , Delbeek, J. C. , DeVantier, L. , Edgar, G. J. , Edwards, A. J. , Fenner, D. , Guzmán, H. M. , Hoeksema, B. W. , Hodgson, G. , Johan, O. , Licuanan, W. Y. , Livingstone, S. R. , … Wood, E. (2008). One‐third of reef‐building corals face elevated extinction risk from climate change and local impacts. Science, 321(5888), 560–563. 10.1126/science.1159196 18653892

[ece39096-bib-0007] Carvalho, G. R. (1994). Genetics of aquatic clonal organisms. In: Beaumont A (Ed.). Genetics andevolution of aquatic organisms (Beaumont, pp. 291–323). Chapman and Hall.

[ece39096-bib-0008] Catchen, J. , Hohenlohe, P. A. , Bassham, S. , Amores, A. , & Cresko, W. A. (2013). Stacks: An analysis tool set for population genomics. Molecular Ecology, 22(11), 3124–3140. 10.1111/mec.12354 23701397 PMC3936987

[ece39096-bib-0009] Cerca, J. , Maurstad, M. F. , Rochette, N. C. , Rivera‐Colón, A. G. , Rayamajhi, N. , Catchen, J. M. , & Struck, T. H. (2021). Removing the bad apples: A simple bioinformatic method to improve loci‐recovery in de novo RADseq data for non‐model organisms. Methods in Ecology and Evolution, 12(5), 805–817. 10.1111/2041-210X.13562

[ece39096-bib-0010] Cousyn, C. , Meester, L. D. , Colbourne, J. K. , Brendonck, L. , Verschuren, D. , & Volckaert, F. (2001). Rapid, local adaptation of zooplankton behavior to changes in predation pressure in the absence of neutral genetic changes. Proceedings of the National Academy of Sciences, 98(11), 6256–6260. 10.1073/pnas.111606798 PMC33455 11353872

[ece39096-bib-0011] Csardi, G. , & Nepusz, T. (2005). The igraph software package for complex network research. InterJournal, Complex Systems, 1695(5), 1–9.

[ece39096-bib-0012] Danecek, P. , Auton, A. , Abecasis, G. , Albers, C. A. , Banks, E. , DePristo, M. A. , Handsaker, R. E. , Lunter, G. , Marth, G. T. , Sherry, S. T. , McVean, G. , Durbin, R. , & 1000 Genomes Project Analysis Group . (2011). The variant call format and VCFtools. Bioinformatics, 27(15), 2156–2158. 10.1093/bioinformatics/btr330 21653522 PMC3137218

[ece39096-bib-0013] De Meester, L. (1996). Local genetic differentiation and adaptation in freshwater zooplankton populations: Patterns and processes. Écoscience, 3(4), 385–399. 10.1080/11956860.1996.11682356

[ece39096-bib-0014] De Meester, L. , Gomez, A. , & Simon, J. C. (2004). Evolutionary and ecological genetics of cyclical parthenogens (pp. 122–134). Oxford University Press. https://lirias.kuleuven.be/1704928

[ece39096-bib-0015] De Meester, L. , Vanoverbeke, J. , De Gelas, K. , Ortells, R. , & Spaak, P. (2006). Genetic structure of cyclic parthenogenetic zooplankton populations – A conceptual framework. Archiv für Hydrobiologie, 167(1–4), 217–244. 10.1127/0003-9136/2006/0167-0217

[ece39096-bib-0016] Decaestecker, E. , De Meester, L. , & Mergeay, J. (2009). Cyclical parthenogenesis in daphnia: Sexual versus asexual reproduction. In I. Schön , K. Martens , & P. Dijk (Eds.), Lost sex (pp. 295–316). Springer Netherlands. 10.1007/978-90-481-2770-2_15

[ece39096-bib-0017] Deng, H.‐W. , & Lynch, M. (1996). Change of genetic architecture in response to sex. Genetics, 143(1), 203–212. 8722775 10.1093/genetics/143.1.203 PMC1207254

[ece39096-bib-0018] Dorken, M. E. , & Eckert, C. G. (2001). Severely reduced sexual reproduction in northern populations of a clonal plant, Decodonverticillatus (Lythraceae). Journal of Ecology, 89(3), 339–350. 10.1046/j.1365-2745.2001.00558.x

[ece39096-bib-0019] Fraune, S. , Anton‐Erxleben, F. , Augustin, R. , Franzenburg, S. , Knop, M. , Schröder, K. , Willoweit‐Ohl, D. , & Bosch, T. C. (2015). Bacteria–bacteria interactions within the microbiota of the ancestral metazoan hydra contribute to fungal resistance. The ISME Journal, 9(7), 1543–1556. 10.1038/ismej.2014.239 25514534 PMC4478695

[ece39096-bib-0020] Goudet, J. , Jombart, T. , Kamvar, Z. N. , Archer, E. , & Hardy, O. (2020). *Hierfstat: Estimation and tests of hierarchical F‐statistics* (0.5‐7) [computer software]. https://CRAN.R‐project.org/package=hierfstat

[ece39096-bib-0021] Hadany, L. , & Otto, S. P. (2007). The evolution of condition‐dependent sex in the face of high costs. Genetics, 176(3), 1713–1727. 10.1534/genetics.107.074203 17483405 PMC1931531

[ece39096-bib-0022] Hairston, N. G. , Holtmeier, C. L. , Lampert, W. , Weider, L. J. , Post, D. M. , Fischer, J. M. , Cáceres, C. E. , Fox, J. A. , & Gaedke, U. (2001). Natural selection for grazer resistance to toxic cyanobacteria: Evolution of phenotypic plasticity? Evolution, 55(11), 2203–2214. 10.1111/j.0014-3820.2001.tb00736.x 11794781

[ece39096-bib-0023] Halkett, F. , Simon, J.‐C. , & Balloux, F. (2005). Tackling the population genetics of clonal and partially clonal organisms. Trends in Ecology and Evolution, 20(4), 194–201. 10.1016/j.tree.2005.01.001 16701368

[ece39096-bib-0024] Hoffmann, A. A. , Hallas, R. J. , Dean, J. A. , & Schiffer, M. (2003). Low potential for climatic stress adaptation in a rainforest drosophila species. Science (New York, N.Y.), 301(5629), 100–102. 10.1126/science.1084296 12843394

[ece39096-bib-0025] Jombart, T. (2008). Adegenet: A R package for the multivariate analysis of genetic markers. Bioinformatics, 24(11), 1403–1405. 10.1093/bioinformatics/btn129 18397895

[ece39096-bib-0026] Jombart, T. , Devillard, S. , & Balloux, F. (2010). Discriminant analysis of principal components: A new method for the analysis of genetically structured populations. BMC Genetics, 11(1), 94. 10.1186/1471-2156-11-94 20950446 PMC2973851

[ece39096-bib-0027] Jones, O. R. , & Wang, J. (2010). COLONY: A program for parentage and sibship inference from multilocus genotype data. Molecular Ecology Resources, 10(3), 551–555. 10.1111/j.1755-0998.2009.02787.x 21565056

[ece39096-bib-0028] Kamvar, Z. N. , Tabima, J. F. , & Grünwald, N. J. (2014). *Poppr*: An R package for genetic analysis of populations with clonal, partially clonal, and/or sexual reproduction. PeerJ, 2, e281. 10.7717/peerj.281 24688859 PMC3961149

[ece39096-bib-0029] King, C. E. , & Schonfeld, J. (2001). The approach to equilibrium of multilocus genotype diversity under clonal selection and cyclical parthenogenesis. In L. Sanoamuang , H. Segers , R. J. Shiel , & R. D. Gulati (Eds.), Rotifera IX (pp. 323–331). Springer Netherlands. 10.1007/978-94-010-0756-6_42

[ece39096-bib-0030] Kokko, H. (2020). When synchrony makes the best of both worlds even better: How well do we really understand facultative sex? The American Naturalist, 195(2), 380–392. 10.1086/706812 32017623

[ece39096-bib-0031] Langer, G. , Nehrke, G. , Probert, I. , Ly, J. , & Ziveri, P. (2009). Strain‐specific responses of *Emiliania huxleyi* to changing seawater carbonate chemistry. Biogeosciences, 6(11), 2637–2646. 10.5194/bg-6-2637-2009

[ece39096-bib-0032] Lenhoff, H. M. (1983). Hydra: Research methods. Plenum Press.

[ece39096-bib-0033] Lüdecke, D. , Waggoner, P. D. , & Makowski, D. (2019). Insight: A unified Interface to access information from model objects in R. Journal of Open Source Software, 4(38), 1412. 10.21105/joss.01412

[ece39096-bib-0034] Lynch, M. , & Gabriel, W. (1983). Phenotypic evolution and parthenogenesis. The American Naturalist, 122(6), 745–764.

[ece39096-bib-0035] Miklós, M. , Laczkó, L. , Sramkó, G. , Sebestyén, F. , Barta, Z. , & Tökölyi, J. (2021). Phenotypic plasticity rather than genotype drives reproductive choices in hydra populations. Molecular Ecology, 30(5), 1206–1222. 10.1111/mec.15810 33465828

[ece39096-bib-0036] Ngo, K. S. , R‐Almási, B. , Barta, Z. , & Tökölyi, J. (2021). Experimental manipulation of body size alters life history in hydra. Ecology Letters, 24(4), 728–738. 10.1111/ele.13698 33606896

[ece39096-bib-0037] Nunney, L. (1989). The maintenance of sex by group selection. Evolution, 43(2), 245–257. 10.1111/j.1558-5646.1989.tb04225.x 28568559

[ece39096-bib-0038] Ortells, R. , Gómez, A. , & Serra, M. (2006). Effects of duration of the planktonic phase on rotifer genetic diversity. Archiv für Hydrobiologie, 167(1–4), 203–216. 10.1127/0003-9136/2006/0167-0203

[ece39096-bib-0039] Paradis, E. (2010). Pegas: An R package for population genetics with an integrated‐modular approach. Bioinformatics, 26(3), 419–420. 10.1093/bioinformatics/btp696 20080509

[ece39096-bib-0040] Paris, J. R. , Stevens, J. R. , & Catchen, J. M. (2017). Lost in parameter space: a road map for STACKS. Methods in Ecology and Evolution, 8(10), 1360–1373. 10.1111/2041-210X.12775

[ece39096-bib-0041] Pfrender, M. E. , & Lynch, M. (2000). Quantitative genetic variation in daphnia: Temporal changes in genetic architecture. Evolution, 54(5), 1502–1509. 10.1111/j.0014-3820.2000.tb00696.x 11108579

[ece39096-bib-0042] Pistevos, J. C. A. , Calosi, P. , Widdicombe, S. , & Bishop, J. D. D. (2011). Will variation among genetic individuals influence species responses to global climate change? Oikos, 120(5), 675–689. 10.1111/j.1600-0706.2010.19470.x

[ece39096-bib-0043] Polidoro, B. A. , Carpenter, K. E. , Collins, L. , Duke, N. C. , Ellison, A. M. , Ellison, J. C. , Farnsworth, E. J. , Fernando, E. S. , Kathiresan, K. , Koedam, N. E. , Livingstone, S. R. , Miyagi, T. , Moore, G. E. , Nam, V. N. , Ong, J. E. , Primavera, J. H. , Iii, S. G. S. , Sanciangco, J. C. , Sukardjo, S. , … Yong, J. W. H. (2010). The loss of species: Mangrove extinction risk and geographic areas of global concern. PLoS One, 5(4), e10095. 10.1371/journal.pone.0010095 20386710 PMC2851656

[ece39096-bib-0044] R Core Team . (2020). R: A language and environment for statistical computing. R Foundation for Statistical Computing.

[ece39096-bib-0045] Reisa, J. (1973). Ecology of hydra. In A. Burnett (Ed.), Biology of hydra. Academic Press.

[ece39096-bib-0046] Ribi, G. , Tardent, R. , & Scascighini, C. (1985). Dynamics of hydra populations in Lake Ziirich, Switzerland, and Lake Maggiore, Italy. Swiss Journal of Hydrology, 47(1), 45–56. 10.1007/BF02538183

[ece39096-bib-0047] Rozenfeld, A. F. , Arnaud‐Haond, S. , Hernández‐García, E. , Eguíluz, V. M. , Matías, M. A. , Serrão, E. , & Duarte, C. M. (2007). Spectrum of genetic diversity and networks of clonal organisms. Journal of the Royal Society Interface, 4(17), 1093–1102. 10.1098/rsif.2007.0230 17472906 PMC2396204

[ece39096-bib-0048] Schröder, T. (2005). Diapause in monogonont rotifers. In A. Herzig , R. D. Gulati , C. D. Jersabek , & L. May (Eds.), Rotifera X: Rotifer research: Trends, new tools and recent advances, proceedings of the Xth international rotifer symposium, held in Illmitz, Austria, 7–13 June 2003 (pp. 291–306). Springer Netherlands. 10.1007/1-4020-4408-9_30

[ece39096-bib-0049] Schuchert, P. (2010). The European athecate hydroids and their medusae (hydrozoa, cnidaria): Capitata part 2. Revue Suisse de Zoologie, 117, 337–555.

[ece39096-bib-0050] Sebestyén, F. , Barta, Z. , & Tökölyi, J. (2018). Reproductive mode, stem cells and regeneration in a freshwater cnidarian with postreproductive senescence. Functional Ecology, 32(11), 2497–2508. 10.1111/1365-2435.13189

[ece39096-bib-0051] Sherrill‐Mix, S. (2019). *Taxonomizr: Functions to Work with NCBI Accessions and Taxonomy* (0.5.3) [Computer software]. https://CRAN.R‐project.org/package=taxonomizr

[ece39096-bib-0052] Simon, J.‐C. , Rispe, C. , & Sunnucks, P. (2002). Ecology and evolution of sex in aphids. Trends in Ecology and Evolution, 17(1), 34–39. 10.1016/S0169-5347(01)02331-X

[ece39096-bib-0053] Simons, A. M. (2009). Fluctuating natural selection accounts for the evolution of diversification bet hedging. Proceedings of the Royal Society B: Biological Sciences, 276(1664), 1987–1992. 10.1098/rspb.2008.1920 PMC2677257 19324774

[ece39096-bib-0054] Steele, R. E. , Updegrove, M. D. , Kirolos, S. A. , Mowery, L. , Martínez, D. E. , & Bryant, P. J. (2019). Reproductive bet‐hedging and existence in vernal pools as components of hydra life history. The Biological Bulletin, 237(2), 111–118. 10.1086/705161 31714853

[ece39096-bib-0055] Stelzer, C.‐P. (2012). Population regulation in sexual and asexual rotifers: An eco‐evolutionary feedback to population size? Functional Ecology, 26(1), 180–188. 34764531 10.1111/j.1365-2435.2011.01918.x PMC7611971

[ece39096-bib-0056] Stelzer, C.‐P. , & Lehtonen, J. (2016). Diapause and maintenance of facultative sexual reproductive strategies. Philosophical Transactions of the Royal Society B: Biological Sciences, 371(1706), 20150536. 10.1098/rstb.2015.0536 PMC5031621 27619700

[ece39096-bib-0057] Tarazona, E. , García‐Roger, E. M. , & Carmona, M. J. (2017). Experimental evolution of bet hedging in rotifer diapause traits as a response to environmental unpredictability. Oikos, 126(8), 1162–1172. 10.1111/oik.04186

[ece39096-bib-0058] Tessier, A. J. , & Caceres, C. E. (2004). Differentiation in sex investment by clones and populations of daphnia. Ecology Letters, 7(8), 695–703. 10.1111/j.1461-0248.2004.00627.x

[ece39096-bib-0059] Tessier, A. J. , Young, A. , & Leibold, M. (1992). Population dynamics and body‐size selection in daphnia. Limnology and Oceanography, 37(1), 1–13. 10.4319/lo.1992.37.1.0001

[ece39096-bib-0060] Thorson, J. L. M. , Smithson, M. , Beck, D. , Sadler‐Riggleman, I. , Nilsson, E. , Dybdahl, M. , & Skinner, M. K. (2017). Epigenetics and adaptive phenotypic variation between habitats in an asexual snail. Scientific Reports, 7(1), 14139. 10.1038/s41598-017-14673-6 29074962 PMC5658341

[ece39096-bib-0061] Tökölyi, J. , Gergely, R. , & Miklós, M. (2021). Seasonal variation in sexual readiness in a facultatively sexual freshwater cnidarian with diapausing eggs. Ecosphere, 12(8), e03713. 10.1002/ecs2.3713

[ece39096-bib-0062] Tökölyi, J. , Ősz, Z. , Sebestyén, F. , & Barta, Z. (2017). Resource allocation and post‐reproductive degeneration in the freshwater cnidarian *Hydra oligactis* (Pallas, 1766). Zoology, 120, 110–116. 10.1016/j.zool.2016.06.009 27491450

[ece39096-bib-0063] Tomczyk, S. , Fischer, K. , Austad, S. , & Galliot, B. (2015). Hydra, a powerful model for aging studies. Invertebrate Reproduction and Development, 59(sup1), 11–16. 10.1080/07924259.2014.927805 26120246 PMC4463768

[ece39096-bib-0064] Walsh, E. J. , Smith, H. A. , & Wallace, R. L. (2014). Rotifers of temporary waters. International Review of Hydrobiology, 99(1–2), 3–19. 10.1002/iroh.201301700

[ece39096-bib-0065] Wang, J. (2016). Individual identification from genetic marker data: Developments and accuracy comparisons of methods. Molecular Ecology Resources, 16(1), 163–175. 10.1111/1755-0998.12452 26230747

[ece39096-bib-0066] Waycott, M. , Duarte, C. M. , Carruthers, T. J. B. , Orth, R. J. , Dennison, W. C. , Olyarnik, S. , Calladine, A. , Fourqurean, J. W. , Heck, K. L. , Hughes, A. R. , Kendrick, G. A. , Kenworthy, W. J. , Short, F. T. , & Williams, S. L. (2009). Accelerating loss of seagrasses across the globe threatens coastal ecosystems. Proceedings of the National Academy of Sciences, 106(30), 12377–12381. 10.1073/pnas.0905620106 PMC2707273 19587236

[ece39096-bib-0067] Welch, P. S. , & Loomis, H. A. (1924). A limnological study of *Hydra oligactis* in Douglas Lake, Michigan. Transactions of the American Microscopical Society, 43(4), 203. 10.2307/3221738

[ece39096-bib-0068] Yin, M. , Wolinska, J. , & Gießler, S. (2010). Clonal diversity, clonal persistence and rapid taxon replacement in natural populations of species and hybrids of the Daphnia longispina complex. Molecular Ecology, 19(19), 4168–4178. 10.1111/j.1365-294X.2010.04807.x 20819161

