# Supplementary material for: Seasonal variation of genotypes and reproductive plasticity in a facultative clonal freshwater invertebrate animal (Hydra oligactis) living in a temperate lake
Source: Ecol Evol. 2022 Jul 14;12(7):e9096. doi: 10.1002/ece3.9096 (PMC9280439; doi:10.1002/ece3.9096)

**Appendix**

Appendix Tables:

Appendix Table 1. Individual ID, Location IDs, Clone ID, Collection dates, GPS coordinates, Sampling occasions, GenBank association numbers of the collection sites of sequenced *H. oligactis* polyps.

| **Individual ID** | **Location ID** | **Clone ID** | **Collection Date** | **Sampling occasion** | **GPS cordinates** | **GenBank association number** |
| --- | --- | --- | --- | --- | --- | --- |
| M28_2018autumn_1_3 | M28/1 | 6 | 2018.10.01 | 2018 Autumn | N47.67121 E20.86296 | SAMN21437818 |
| M28_2018autumn_2_1 | M28/2 | 1 | 2018.10.01 | 2018 Autumn | N47.67130 E20.86312 | SAMN21437819 |
| M28_2018autumn_2_2 | M28/2 | 6 | 2018.10.01 | 2018 Autumn | N47.67130 E20.86312 | SAMN21437820 |
| M28_2018autumn_2_3 | M28/2 | 1 | 2018.10.01 | 2018 Autumn | N47.67130 E20.86312 | SAMN21437821 |
| M28_2018autumn_3_3 | M28/3 | 7 | 2018.10.01 | 2018 Autumn | N47.67112 E20.86335 | SAMN21437822 |
| M28_2018autumn_3_4 | M28/3 | 7 | 2018.10.01 | 2018 Autumn | N47.67112 E20.86335 | SAMN21437823 |
| M28_2018autumn_4_1 | M28/4 | 6 | 2018.10.01 | 2018 Autumn | N47.67118 E20.86389 | SAMN21437824 |
| M28_2018autumn_4_4 | M28/4 | 6 | 2018.10.01 | 2018 Autumn | N47.67118 E20.86389 | SAMN21437825 |
| M28_2018autumn_5_1 | M28/5 | 8 | 2018.10.01 | 2018 Autumn | N47.67116 E20.86399 | SAMN21437826 |
| M28_2018autumn_5_2 | M28/5 | 9 | 2018.10.01 | 2018 Autumn | N47.67116 E20.86399 | SAMN21437827 |
| M28_2018autumn_5_3 | M28/5 | 6 | 2018.10.01 | 2018 Autumn | N47.67116 E20.86399 | SAMN21437828 |
| M28_2018autumn_5_4 | M28/5 | 6 | 2018.10.01 | 2018 Autumn | N47.67116 E20.86399 | SAMN21437829 |
| M28_2018autumn_6_1 | M28/6 | 6 | 2018.10.01 | 2018 Autumn | N47.67113 E20.86446 | SAMN21437830 |
| M28_2018autumn_6_2 | M28/6 | 6 | 2018.10.01 | 2018 Autumn | N47.67113 E20.86446 | SAMN21437831 |
| M28_2018autumn_6_3 | M28/6 | 6 | 2018.10.01 | 2018 Autumn | N47.67113 E20.86446 | SAMN21437832 |
| M28_2018autumn_6_4 | M28/6 | 6 | 2018.10.01 | 2018 Autumn | N47.67113 E20.86446 | SAMN21437833 |
| M28_2018autumn_7_1 | M28/7 | 10 | 2018.10.01 | 2018 Autumn | N47.67105 E20.86687 | SAMN21437834 |
| M28_2018autumn_7_2 | M28/7 | 10 | 2018.10.01 | 2018 Autumn | N47.67105 E20.86687 | SAMN21437835 |
| M28_2018autumn_7_3 | M28/7 | 11 | 2018.10.01 | 2018 Autumn | N47.67105 E20.86687 | SAMN21437836 |
| M28_2018autumn_7_4 | M28/7 | 11 | 2018.10.01 | 2018 Autumn | N47.67105 E20.86687 | SAMN21437837 |
| M28_2018autumn_8_2 | M28/8 | 6 | 2018.10.01 | 2018 Autumn | N47.67091 E20.86738 | SAMN21437838 |
| M28_2018autumn_8_3 | M28/8 | 6 | 2018.10.01 | 2018 Autumn | N47.67091 E20.86738 | SAMN21437839 |
| M28_2018autumn_9_1 | M28/9 | 6 | 2018.10.01 | 2018 Autumn | N47.67107 E20.86776 | SAMN21437840 |
| M28_2018autumn_9_3 | M28/9 | 6 | 2018.10.01 | 2018 Autumn | N47.67107 E20.86776 | SAMN21437841 |
| M28_2018autumn_10_3 | M28/10 | 1 | 2018.10.01 | 2018 Autumn | N47.67151 E20.86570 | SAMN21437843 |
| M28_2018autumn_10_4 | M28/10 | 2 | 2018.10.01 | 2018 Autumn | N47.67151 E20.86570 | SAMN21437844 |
| M28_2018autumn11_1 | M28/11 | 3 | 2018.10.01 | 2018 Autumn | N47.67155 E20.86566 | SAMN21437845 |
| M28_2018autumn_11_2 | M28/11 | 4 | 2018.10.01 | 2018 Autumn | N47.67155 E20.86566 | SAMN21437846 |
| M28_2018autumn_11_3 | M28/11 | 3 | 2018.10.01 | 2018 Autumn | N47.67155 E20.86566 | SAMN21437847 |
| M28_2018autumn_11_4 | M28/11 | 5 | 2018.10.01 | 2018 Autumn | N47.67155 E20.86566 | SAMN21437848 |
| M28_2018spring_1_1 | M28/1 | 14 | 2018.05.31 | 2018 Spring | N47.67133 E20.86300 | SAMN21437849 |
| M28_2018spring_1_2 | M28/1 | 15 | 2018.05.31 | 2018 Spring | N47.67133 E20.86300 | SAMN21437850 |
| M28_2018spring_1_3 | M28/1 | 17 | 2018.05.31 | 2018 Spring | N47.67133 E20.86300 | SAMN21437851 |
| M28_2018spring_2_1 | M28/2 | 13 | 2018.05.31 | 2018 Spring | N47.67136 E20.86285 | SAMN21437852 |
| M28_2018spring_2_2 | M28/2 | 13 | 2018.05.31 | 2018 Spring | N47.67136 E20.86285 | SAMN21437853 |
| M28_2018spring_3_1 | M28/3 | 25 | 2018.05.31 | 2018 Spring | N47.67126 E20.86316 | SAMN21437854 |
| M28_2018spring_3_4 | M28/3 | 26 | 2018.05.31 | 2018 Spring | N47.67126 E20.86316 | SAMN21437855 |
| M28_2018spring_4_2 | M28/4 | 1 | 2018.05.31 | 2018 Spring | N47.67111 E20.86307 | SAMN21437856 |
| M28_2018spring_4_3 | M28/4 | 18 | 2018.05.31 | 2018 Spring | N47.67111 E20.86307 | SAMN21437857 |
| M28_2018spring_5_2 | M28/5 | 11 | 2018.05.31 | 2018 Spring | N47.67120 E20.86340 | SAMN21437858 |
| M28_2018spring_5_3 | M28/5 | 18 | 2018.05.31 | 2018 Spring | N47.67120 E20.86340 | SAMN21437859 |
| M28_2018spring_6_1 | M28/6 | 20 | 2018.05.31 | 2018 Spring | N47.67116 E20.86344 | SAMN21437860 |
| M28_2018spring_6_2 | M28/6 | 27 | 2018.05.31 | 2018 Spring | N47.67116 E20.86344 | SAMN21437861 |
| M28_2018spring_6_3 | M28/6 | 20 | 2018.05.31 | 2018 Spring | N47.67116 E20.86344 | SAMN21437862 |
| M28_2018spring_7_1 | M28/7 | 26 | 2018.05.31 | 2018 Spring | N47.67116 E20.86386 | SAMN21437863 |
| M28_2018spring_7_2 | M28/7 | 28 | 2018.05.31 | 2018 Spring | N47.67116 E20.86386 | SAMN21437864 |
| M28_2018spring_7_3 | M28/7 | 29 | 2018.05.31 | 2018 Spring | N47.67116 E20.86386 | SAMN21437865 |
| M28_2018spring_8_2 | M28/8 | 26 | 2018.05.31 | 2018 Spring | N47.67113 E20.86397 | SAMN21437866 |
| M28_2018spring_8_3 | M28/8 | 30 | 2018.05.31 | 2018 Spring | N47.67113 E20.86397 | SAMN21437867 |
| M28_2018spring_9_1 | M28/9 | 31 | 2018.05.31 | 2018 Spring | N47.67112 E20.86415 | SAMN21437868 |
| M28_2018spring_9_2 | M28/9 | 31 | 2018.05.31 | 2018 Spring | N47.67112 E20.86415 | SAMN21437869 |
| M28_2018spring_9_3 | M28/9 | 11 | 2018.05.31 | 2018 Spring | N47.67112 E20.86415 | SAMN21437870 |
| M28_2018spring_10_2 | M28/10 | 12 | 2018.05.31 | 2018 Spring | N47.67115 E20.86458 | SAMN21437871 |
| M28_2018spring_10_3 | M28/10 | 12 | 2018.05.31 | 2018 Spring | N47.67115 E20.86458 | SAMN21437872 |
| M28_2018spring_11_1 | M28/11 | 13 | 2018.05.31 | 2018 Spring | N47.67082 E20.86591 | SAMN21437873 |
| M28_2018spring_12_1 | M28/12 | 15 | 2018.05.31 | 2018 Spring | N47.67101 E20.86592 | SAMN21437874 |
| M28_2018spring_13_1 | M28/13 | 16 | 2018.05.31 | 2018 Spring | N47.67109 E20.86591 | SAMN21437875 |
| M28_2018spring_13_2 | M28/13 | 11 | 2018.05.31 | 2018 Spring | N47.67109 E20.86591 | SAMN21437876 |
| M28_2018spring_14_1 | M28/14 | 13 | 2018.05.31 | 2018 Spring | N47.67119 E20.86590 | SAMN21437877 |
| M28_2018spring_14_2 | M28/14 | 18 | 2018.05.31 | 2018 Spring | N47.67119 E20.86590 | SAMN21437878 |
| M28_2018spring_14_3 | M28/14 | 15 | 2018.05.31 | 2018 Spring | N47.67119 E20.86590 | SAMN21437879 |
| M28_2018spring_15_2 | M28/15 | 16 | 2018.05.31 | 2018 Spring | N47.67134 E20.86593 | SAMN21437880 |
| M28_2018spring_15_3 | M28/15 | 19 | 2018.05.31 | 2018 Spring | N47.67134 E20.86593 | SAMN21437881 |
| M28_2018spring_16_2 | M28/16 | 20 | 2018.05.31 | 2018 Spring | N47.67101 E20.86648 | SAMN21437882 |
| M28_2018spring_16_3 | M28/16 | 21 | 2018.05.31 | 2018 Spring | N47.67101 E20.86648 | SAMN21437883 |
| M28_2018spring_17_1 | M28/17 | 22 | 2018.05.31 | 2018 Spring | N47.67098 E20.86695 | SAMN21437884 |
| M28_2018spring_17_2 | M28/17 | 23 | 2018.05.31 | 2018 Spring | N47.67098 E20.86695 | SAMN21437885 |
| M28_2018spring_18_2 | M28/18 | 24 | 2018.05.31 | 2018 Spring | N47.67083 E20.86577 | SAMN21437886 |
| M28_2018spring_18_3 | M28/18 | 15 | 2018.05.31 | 2018 Spring | N47.67083 E20.86577 | SAMN21437887 |
| M28_2018spring_19_2 | M28/19 | 11 | 2018.05.31 | 2018 Spring | N47.67099 E20.86771 | SAMN21437888 |
| M28_2019autumn_1_1 | M28/1 | 34 | 2019.09.24 | 2019 Autumn | N47.67070 E20.86588 | SAMN21437889 |
| M28_2019autumn_1_2 | M28/1 | 37 | 2019.09.24 | 2019 Autumn | N47.67070 E20.86588 | SAMN21437890 |
| M28_2019autumn_1_4 | M28/1 | 38 | 2019.09.24 | 2019 Autumn | N47.67070 E20.86588 | SAMN21437891 |
| M28_2019autumn_1_5 | M28/1 | 39 | 2019.09.24 | 2019 Autumn | N47.67070 E20.86588 | SAMN21437892 |
| M28_2019autumn_4_2 | M28/4 | 40 | 2019.09.24 | 2019 Autumn | N47.67110 E20.86570 | SAMN21437893 |
| M28_2019autumn_4_5 | M28/4 | 41 | 2019.09.24 | 2019 Autumn | N47.67110 E20.86570 | SAMN21437894 |
| M28_2019autumn_5_1 | M28/5 | 42 | 2019.09.24 | 2019 Autumn | N47.67123 E20.86589 | SAMN21437895 |
| M28_2019autumn_5_2 | M28/5 | 29 | 2019.09.24 | 2019 Autumn | N47.67123 E20.86589 | SAMN21437896 |
| M28_2019autumn_5_4 | M28/5 | 42 | 2019.09.24 | 2019 Autumn | N47.67123 E20.86589 | SAMN21437897 |
| M28_2019autumn_6_1 | M28/6 | 6 | 2019.09.24 | 2019 Autumn | N47.67138 E20.86588 | SAMN21437898 |
| M28_2019autumn_6_2 | M28/6 | 43 | 2019.09.24 | 2019 Autumn | N47.67138 E20.86588 | SAMN21437899 |
| M28_2019autumn_6_3 | M28/6 | 42 | 2019.09.24 | 2019 Autumn | N47.67138 E20.86588 | SAMN21437900 |
| M28_2019autumn_6_5 | M28/6 | 42 | 2019.09.24 | 2019 Autumn | N47.67138 E20.86588 | SAMN21437901 |
| M28_2019autumn_7_1 | M28/7 | 35 | 2019.09.24 | 2019 Autumn | N47.67149 E20.86570 | SAMN21437902 |
| M28_2019autumn_7_3 | M28/7 | 42 | 2019.09.24 | 2019 Autumn | N47.67149 E20.86570 | SAMN21437903 |
| M28_2019autumn_7_4 | M28/7 | 42 | 2019.09.24 | 2019 Autumn | N47.67149 E20.86570 | SAMN21437904 |
| M28_2019autumn_8_3 | M28/8 | 32 | 2019.09.24 | 2019 Autumn | N47.67082 E20.86580 | SAMN21437905 |
| M28_2019autumn_8_4 | M28/8 | 32 | 2019.09.24 | 2019 Autumn | N47.67082 E20.86580 | SAMN21437906 |
| M28_2019autumn_9_1 | M28/9 | 44 | 2019.09.24 | 2019 Autumn | N47.67103 E20.86584 | SAMN21437907 |
| M28_2019autumn_9_4 | M28/9 | 45 | 2019.09.24 | 2019 Autumn | N47.67103 E20.86584 | SAMN21437908 |
| M28_2019autumn_10_1 | M28/10 | 32 | 2019.09.24 | 2019 Autumn | N47.67121 E20.86594 | SAMN21437909 |
| M28_2019autumn_10_2 | M28/10 | 32 | 2019.09.24 | 2019 Autumn | N47.67121 E20.86594 | SAMN21437910 |
| M28_2019autumn_10_3 | M28/10 | 33 | 2019.09.24 | 2019 Autumn | N47.67121 E20.86594 | SAMN21437911 |
| M28_2019autumn_10_4 | M28/10 | 32 | 2019.09.24 | 2019 Autumn | N47.67121 E20.86594 | SAMN21437912 |
| M28_2019autumn_11_2 | M28/11 | 35 | 2019.09.24 | 2019 Autumn | N47.67138 E20.86588 | SAMN21437913 |
| M28_2019autumn_11_3 | M28/11 | 35 | 2019.09.24 | 2019 Autumn | N47.67138 E20.86588 | SAMN21437914 |
| M28_2019autumn_12_1 | M28/12 | 36 | 2019.09.24 | 2019 Autumn | N47.67113 E20.86642 | SAMN21437915 |
| M28_2019autumn_12_2 | M28/12 | 36 | 2019.09.24 | 2019 Autumn | N47.67113 E20.86642 | SAMN21437916 |
| M28_2019autumn_13_1 | M28/13 | 33 | 2019.09.24 | 2019 Autumn | N47.67107 E20.86780 | SAMN21437917 |
| M28_2019autumn_13_3 | M28/13 | 11 | 2019.09.24 | 2019 Autumn | N47.67107 E20.86780 | SAMN21437918 |
| M28_2019spring_1_3 | M28/1 | 6 | 2019.05.16 | 2019 Spring | N47.67120 E20.86294 | SAMN21437919 |
| M28_2019spring_2_1 | M28/2 | 52 | 2019.05.16 | 2019 Spring | N47.67119 E20.86292 | SAMN21437920 |
| M28_2019spring_3_1 | M28/3 | 15 | 2019.05.16 | 2019 Spring | N47.67120 E20.86305 | SAMN21437921 |
| M28_2019spring_3_2 | M28/3 | 6 | 2019.05.16 | 2019 Spring | N47.67120 E20.86305 | SAMN21437922 |
| M28_2019spring_4_1 | M28/4 | 15 | 2019.05.16 | 2019 Spring | N47.67115 E20.86312 | SAMN21437923 |
| M28_2019spring_5_1 | M28/5 |  | 2019.05.16 | 2019 Spring | N47.67110 E20.86330 | SAMN21437924 |
| M28_2019spring_5_2 | M28/5 | 15 | 2019.05.16 | 2019 Spring | N47.67110 E20.86330 | SAMN21437925 |
| M28_2019spring_5_3 | M28/5 |  | 2019.05.16 | 2019 Spring | N47.67110 E20.86330 | SAMN21437926 |
| M28_2019spring_6_2 | M28/6 | 26 | 2019.05.16 | 2019 Spring | N47.67105 E20.86331 | SAMN21437927 |
| M28_2019spring_6_3 | M28/6 |  | 2019.05.16 | 2019 Spring | N47.67105 E20.86331 | SAMN21437928 |
| M28_2019spring_7_1 | M28/7 | 6 | 2019.05.16 | 2019 Spring | N47.67110 E20.86379 | SAMN21437929 |
| M28_2019spring_8_2 | M28/8 | 53 | 2019.05.16 | 2019 Spring | N47.67103 E20.86385 | SAMN21437930 |
| M28_2019spring_9_2 | M28/9 | 15 | 2019.05.16 | 2019 Spring | N47.67103 E20.86402 | SAMN21437931 |
| M28_2019spring_9_3 | M28/9 | 6 | 2019.05.16 | 2019 Spring | N47.67103 E20.86402 | SAMN21437932 |
| M28_2019spring_10_3 | M28/10 | 46 | 2019.05.16 | 2019 Spring | N47.67111 E20.86420 | SAMN21437933 |
| M28_2019spring_11_1 | M28/11 | 6 | 2019.05.16 | 2019 Spring | N47.67112 E20.86434 | SAMN21437934 |
| M28_2019spring_11_2 | M28/11 | 15 | 2019.05.16 | 2019 Spring | N47.67112 E20.86434 | SAMN21437935 |
| M28_2019spring_13_2 | M28/13 |  | 2019.05.16 | 2019 Spring | N47.67106 E20.86496 | SAMN21437936 |
| M28_2019spring_13_3 | M28/13 | 20 | 2019.05.16 | 2019 Spring | N47.67106 E20.86496 | SAMN21437937 |
| M28_2019spring_14_1 | M28/14 | 11 | 2019.05.16 | 2019 Spring | N47.67133 E20.86584 | SAMN21437938 |
| M28_2019spring_14_2 | M28/14 | 47 | 2019.05.16 | 2019 Spring | N47.67133 E20.86584 | SAMN21437939 |
| M28_2019spring_14_3 | M28/14 | 20 | 2019.05.16 | 2019 Spring | N47.67133 E20.86584 | SAMN21437940 |
| M28_2019spring_15_1 | M28/15 |  | 2019.05.16 | 2019 Spring | N47.67078 E20.86577 | SAMN21437941 |
| M28_2019spring_15_2 | M28/15 | 11 | 2019.05.16 | 2019 Spring | N47.67078 E20.86577 | SAMN21437942 |
| M28_2019spring_15_3 | M28/15 | 6 | 2019.05.16 | 2019 Spring | N47.67078 E20.86577 | SAMN21437943 |
| M28_2019spring_16_1 | M28/16 | 48 | 2019.05.16 | 2019 Spring | N47.67079 E20.86584 | SAMN21437944 |
| M28_2019spring_16_2 | M28/16 | 15 | 2019.05.16 | 2019 Spring | N47.67079 E20.86584 | SAMN21437945 |
| M28_2019spring_16_3 | M28/16 |  | 2019.05.16 | 2019 Spring | N47.67079 E20.86584 | SAMN21437946 |
| M28_2019spring_17_1 | M28/17 | 15 | 2019.05.16 | 2019 Spring | N47.67096 E20.86584 | SAMN21437947 |
| M28_2019spring_17_2 | M28/17 | 6 | 2019.05.16 | 2019 Spring | N47.67096 E20.86584 | SAMN21437948 |
| M28_2019spring_17_3 | M28/17 | 11 | 2019.05.16 | 2019 Spring | N47.67096 E20.86584 | SAMN21437949 |
| M28_2019spring_18_1 | M28/18 | 20 | 2019.05.16 | 2019 Spring | N47.67135 E20.86592 | SAMN21437950 |
| M28_2019spring_18_3 | M28/18 | 49 | 2019.05.16 | 2019 Spring | N47.67135 E20.86592 | SAMN21437951 |
| M28_2019spring_19_1 | M28/19 | 50 | 2019.05.16 | 2019 Spring | N47.67091 E20.86687 | SAMN21437952 |
| M28_2019spring_19_2 | M28/19 | 20 | 2019.05.16 | 2019 Spring | N47.67091 E20.86687 | SAMN21437953 |
| M28_2019spring_19_3 | M28/19 | 20 | 2019.05.16 | 2019 Spring | N47.67091 E20.86687 | SAMN21437954 |
| M28_2019spring_20_1 | M28/20 | 51 | 2019.05.16 | 2019 Spring | N47.67102 E20.86720 | SAMN21437955 |
| M28_2019spring_20_2 | M28/20 | 52 | 2019.05.16 | 2019 Spring | N47.67102 E20.86720 | SAMN21437956 |

Appendix Table 2. Read statistics for each genotyped strain: total number of raw reads (Total), reads that were removed due to missing RAD-tags (NoRadTag) or low quality reads (LowQuality); the number of retained reads after Stacks quality filtering (Retained) and decontamination (Uncontaminated); final coverage (Coverage), before removal of PCR duplicates.

| **Strain ID** | **Total** | **NoRadTag** | **LowQuality** | **Retained** | **Uncont.reads** | **Coverage** |
| --- | --- | --- | --- | --- | --- | --- |
| M28_2018autumn_1_3 | 11010454 | 62152 | 23531 | 10451026 | 5471282 | 22.15x |
| M28_2018autumn_10_3 | 6881452 | 34036 | 14388 | 6543317 | 3509496 | 14.37x |
| M28_2018autumn_10_4 | 5980048 | 61070 | 12867 | 5515046 | 2900414 | 12.88x |
| M28_2018autumn_11_1 | 6657262 | 40965 | 13713 | 6294547 | 3257696 | 12.54x |
| M28_2018autumn_11_2 | 7092256 | 48911 | 15631 | 6505079 | 3134594 | 12.57x |
| M28_2018autumn_11_3 | 8281742 | 54594 | 17408 | 7890379 | 4046782 | 17.22x |
| M28_2018autumn_11_4 | 9717486 | 38069 | 20442 | 9158991 | 4792160 | 18.84x |
| M28_2018autumn_2_1 | 16063724 | 73822 | 33996 | 15102246 | 7921026 | 30.70x |
| M28_2018autumn_2_2 | 15738804 | 83534 | 32803 | 15055102 | 7659246 | 29.83x |
| M28_2018autumn_2_3 | 14894504 | 61311 | 31332 | 14145751 | 7350122 | 27.59x |
| M28_2018autumn_3_3 | 14139620 | 116238 | 29650 | 13078455 | 6831438 | 23.22x |
| M28_2018autumn_3_4 | 14215030 | 60941 | 29492 | 13505195 | 6994260 | 24.11x |
| M28_2018autumn_4_1 | 11255440 | 122068 | 23165 | 10637590 | 5293724 | 20.49x |
| M28_2018autumn_4_4 | 14243908 | 75763 | 29331 | 13535028 | 5695688 | 21.31x |
| M28_2018autumn_5_1 | 7154864 | 71668 | 15048 | 6614373 | 3489582 | 15.21x |
| M28_2018autumn_5_2 | 8108948 | 57721 | 16903 | 7567837 | 3994440 | 16.02x |
| M28_2018autumn_5_3 | 6502572 | 78816 | 13395 | 6094976 | 3218870 | 13.17x |
| M28_2018autumn_5_4 | 9711134 | 32625 | 11495 | 9195520 | 3238718 | 12.51x |
| M28_2018autumn_6_1 | 8406550 | 49639 | 17823 | 7979630 | 4231938 | 17.02x |
| M28_2018autumn_6_2 | 7382872 | 48337 | 15794 | 6983405 | 3648430 | 15.67x |
| M28_2018autumn_6_3 | 9018230 | 41592 | 19044 | 8573734 | 4469840 | 18.95x |
| M28_2018autumn_6_4 | 7889564 | 53840 | 10257 | 7459020 | 3516650 | 14.29x |
| M28_2018autumn_7_1 | 7801496 | 82460 | 16306 | 7307963 | 3703290 | 15.79x |
| M28_2018autumn_7_2 | 13330384 | 36043 | 28440 | 12781439 | 6736348 | 25.71x |
| M28_2018autumn_7_3 | 7657342 | 54642 | 16366 | 7197772 | 3887422 | 15.05x |
| M28_2018autumn_7_4 | 8143774 | 40822 | 17278 | 7734644 | 4077672 | 16.39x |
| M28_2018autumn_8_2 | 11467852 | 84715 | 23557 | 10908496 | 5726180 | 22.99x |
| M28_2018autumn_8_3 | 9074130 | 36765 | 19096 | 8711073 | 4330960 | 16.02x |
| M28_2018autumn_9_1 | 6194302 | 45877 | 13122 | 5853716 | 3083030 | 13.39x |
| M28_2018autumn_9_3 | 4910986 | 42175 | 10172 | 4601341 | 2464898 | 9.47x |
| M28_2018spring_1_1 | 6887280 | 81613 | 16694 | 6204390 | 3115978 | 13.40x |
| M28_2018spring_1_2 | 11748200 | 83033 | 24737 | 10992697 | 5742008 | 23.07x |
| M28_2018spring_1_3 | 11011836 | 36194 | 23508 | 10503112 | 5487990 | 21.29x |
| M28_2018spring_10_2 | 7795636 | 34335 | 16263 | 7432912 | 3947810 | 16.73x |
| M28_2018spring_10_3 | 8074628 | 40751 | 12196 | 7510664 | 3611716 | 13.82x |
| M28_2018spring_11_1 | 8865396 | 34430 | 19088 | 8477518 | 4438452 | 18.36x |
| M28_2018spring_12_1 | 10344100 | 49531 | 21595 | 9714870 | 5039450 | 20.97x |
| M28_2018spring_13_1 | 9472126 | 57207 | 20083 | 9015563 | 3941926 | 16.99x |
| M28_2018spring_13_2 | 10431156 | 34988 | 22314 | 9998304 | 5273694 | 21.25x |
| M28_2018spring_14_1 | 8835836 | 53702 | 18298 | 8360292 | 4478776 | 15.25x |
| M28_2018spring_14_2 | 8120416 | 52817 | 14651 | 7467855 | 3600596 | 13.11x |
| M28_2018spring_14_3 | 10982956 | 42510 | 14420 | 10199589 | 4190148 | 15.42x |
| M28_2018spring_15_2 | 7979006 | 47234 | 16635 | 7528737 | 3987078 | 15.48x |
| M28_2018spring_15_3 | 6056312 | 47477 | 12484 | 5734848 | 2930862 | 13.38x |
| M28_2018spring_16_2 | 11404078 | 52124 | 23799 | 10801425 | 5626622 | 21.19x |
| M28_2018spring_16_3 | 8400726 | 87776 | 19220 | 7660348 | 3408884 | 13.91x |
| M28_2018spring_17_1 | 13536412 | 55629 | 28679 | 12966751 | 6765446 | 28.76x |
| M28_2018spring_17_2 | 23575708 | 64024 | 49072 | 22510954 | 11973474 | 19.51x |
| M28_2018spring_18_2 | 12329970 | 144106 | 25102 | 11507711 | 6401976 | 13.18x |
| M28_2018spring_18_3 | 7162522 | 80912 | 13874 | 6458032 | 3202192 | 9.09x |
| M28_2018spring_19_2 | 12367012 | 61365 | 26210 | 11765022 | 6188532 | 24.71x |
| M28_2018spring_2_1 | 8045166 | 44470 | 11360 | 7462002 | 3151906 | 13.60x |
| M28_2018spring_2_2 | 8459952 | 53682 | 17572 | 8043791 | 4095462 | 14.39x |
| M28_2018spring_3_1 | 11177938 | 55624 | 23616 | 10651475 | 5649166 | 21.07x |
| M28_2018spring_3_4 | 12123744 | 74274 | 25797 | 11366661 | 4775718 | 19.02x |
| M28_2018spring_4_2 | 11874054 | 63028 | 25168 | 11196077 | 5860572 | 23.18x |
| M28_2018spring_4_3 | 11757212 | 78744 | 24749 | 11163547 | 5742154 | 23.70x |
| M28_2018spring_5_2 | 7631326 | 40526 | 16340 | 7265928 | 3855362 | 15.64x |
| M28_2018spring_5_3 | 7041192 | 77021 | 14918 | 6498312 | 3482130 | 14.13x |
| M28_2018spring_6_1 | 11640872 | 52978 | 24494 | 11025947 | 5591816 | 21.86x |
| M28_2018spring_6_2 | 9524976 | 68798 | 13012 | 8835259 | 3195762 | 10.91x |
| M28_2018spring_6_3 | 4896872 | 68805 | 10154 | 4534791 | 2364774 | 11.01x |
| M28_2018spring_7_1 | 10663390 | 94417 | 22178 | 10037786 | 5526600 | 12.13x |
| M28_2018spring_7_2 | 6535124 | 63960 | 10430 | 5963551 | 2928964 | 10.91x |
| M28_2018spring_7_3 | 7227316 | 60126 | 16845 | 6606318 | 3303864 | 12.91x |
| M28_2018spring_8_2 | 9030370 | 45472 | 15881 | 8266169 | 3503292 | 13.10x |
| M28_2018spring_8_3 | 8279008 | 36510 | 17500 | 7900803 | 4154822 | 18.30x |
| M28_2018spring_9_1 | 6729394 | 99509 | 15141 | 6062217 | 3144916 | 11.22x |
| M28_2018spring_9_2 | 8803066 | 56878 | 18200 | 8253163 | 4067536 | 11.42x |
| M28_2018spring_9_3 | 7333822 | 46967 | 15201 | 6990343 | 3693828 | 15.39x |
| M28_2019autumn_1_1 | 15930978 | 105194 | 10476 | 15023547 | 6914606 | 23.26x |
| M28_2019autumn_1_2 | 20838572 | 92905 | 13977 | 19754148 | 5120824 | 17.50x |
| M28_2019autumn_1_4 | 11671652 | 56946 | 8077 | 10963566 | 5739128 | 20.63x |
| M28_2019autumn_1_5 | 12450506 | 44672 | 8536 | 11803773 | 6020284 | 20.12x |
| M28_2019autumn_10_1 | 5508592 | 36760 | 3718 | 5243191 | 2753194 | 11.97x |
| M28_2019autumn_10_2 | 10112236 | 42203 | 6830 | 9635137 | 4948020 | 17.72x |
| M28_2019autumn_10_3 | 7676888 | 34766 | 5230 | 7317474 | 3632668 | 12.59x |
| M28_2019autumn_10_4 | 6169408 | 30171 | 4157 | 5860796 | 3097402 | 12.96x |
| M28_2019autumn_11_2 | 9753772 | 68627 | 6502 | 9172564 | 4732244 | 18.37x |
| M28_2019autumn_11_3 | 10272194 | 26091 | 7023 | 9859115 | 4956560 | 18.95x |
| M28_2019autumn_12_1 | 8028240 | 33933 | 5535 | 7582321 | 3991878 | 15.68x |
| M28_2019autumn_12_2 | 11535172 | 47838 | 7724 | 10923723 | 5823588 | 14.90x |
| M28_2019autumn_13_1 | 5826292 | 40805 | 3835 | 5572061 | 2901644 | 10.97x |
| M28_2019autumn_13_3 | 10410564 | 41511 | 6929 | 9966610 | 5351170 | 17.08x |
| M28_2019autumn_4_2 | 15825752 | 98817 | 10647 | 15015103 | 5531930 | 19.69x |
| M28_2019autumn_4_5 | 14891052 | 55101 | 10134 | 14047835 | 7403968 | 22.99x |
| M28_2019autumn_5_1 | 7501716 | 50193 | 5240 | 6995475 | 3766392 | 12.79x |
| M28_2019autumn_5_2 | 7580824 | 31202 | 5222 | 7169172 | 3663120 | 15.03x |
| M28_2019autumn_5_4 | 6913496 | 40405 | 4880 | 6473695 | 3397250 | 14.70x |
| M28_2019autumn_6_1 | 7476752 | 27827 | 5087 | 7095777 | 3674808 | 14.99x |
| M28_2019autumn_6_2 | 9538902 | 37882 | 6581 | 8969307 | 3904298 | 15.70x |
| M28_2019autumn_6_3 | 5443196 | 33009 | 3786 | 5062200 | 2635372 | 11.77x |
| M28_2019autumn_6_5 | 9308670 | 59823 | 6287 | 8787787 | 4574436 | 18.09x |
| M28_2019autumn_7_1 | 9851852 | 42338 | 6664 | 9248707 | 4214204 | 16.17x |
| M28_2019autumn_7_3 | 5905746 | 30514 | 3997 | 5531968 | 2584228 | 11.32x |
| M28_2019autumn_7_4 | 14133466 | 41912 | 9426 | 13356421 | 3541386 | 12.39x |
| M28_2019autumn_8_3 | 7548914 | 49159 | 5267 | 7175841 | 3754442 | 13.62x |
| M28_2019autumn_8_4 | 7706616 | 33570 | 5422 | 7273018 | 3894806 | 13.10x |
| M28_2019autumn_9_1 | 6538086 | 45586 | 4388 | 6152308 | 3057712 | 12.38x |
| M28_2019autumn_9_4 | 7231036 | 34845 | 4886 | 6868143 | 3604770 | 14.80x |
| M28_2019spring_1_3 | 6361298 | 95694 | 16643 | 5692680 | 2730504 | 11.80x |
| M28_2019spring_10_3 | 6999658 | 64424 | 11488 | 6408482 | 2396106 | 10.31x |
| M28_2019spring_11_1 | 6606106 | 34681 | 9549 | 6132563 | 2568904 | 11.73x |
| M28_2019spring_11_2 | 5865694 | 70039 | 22019 | 5148645 | 2311480 | 10.19x |
| M28_2019spring_13_2 | 20554972 | 87320 | 25789 | 18910377 | 1841622 | 7.52x |
| M28_2019spring_13_3 | 6451684 | 67074 | 15012 | 5872852 | 2584404 | 11.07x |
| M28_2019spring_14_1 | 6820084 | 60205 | 13948 | 6152811 | 2843000 | 11.10x |
| M28_2019spring_14_2 | 8417260 | 45906 | 16633 | 7656341 | 3706018 | 14.99x |
| M28_2019spring_14_3 | 8710490 | 34822 | 11629 | 8113246 | 2718742 | 11.38x |
| M28_2019spring_15_1 | 16248214 | 89244 | 16448 | 15307535 | 2081552 | 8.54x |
| M28_2019spring_15_2 | 12597620 | 50832 | 15310 | 11729020 | 2004060 | 8.91x |
| M28_2019spring_15_3 | 6524698 | 61312 | 13880 | 5994653 | 2887552 | 13.17x |
| M28_2019spring_16_1 | 7127910 | 49646 | 12317 | 6544911 | 2692636 | 12.08x |
| M28_2019spring_16_2 | 8968540 | 74248 | 22032 | 7976544 | 3971924 | 14.92x |
| M28_2019spring_16_3 | 12968038 | 85675 | 17460 | 12013716 | 1656784 | 7.60x |
| M28_2019spring_17_1 | 7590410 | 94756 | 32529 | 6567619 | 3143334 | 12.53x |
| M28_2019spring_17_2 | 7537018 | 40006 | 12377 | 6981252 | 2824288 | 11.77x |
| M28_2019spring_17_3 | 8494436 | 89612 | 20323 | 7721597 | 3886398 | 14.73x |
| M28_2019spring_18_1 | 8594018 | 47976 | 15283 | 7914766 | 3603592 | 13.76x |
| M28_2019spring_18_3 | 21154662 | 78104 | 31697 | 19654073 | 3427118 | 11.59x |
| M28_2019spring_19_1 | 9604206 | 53855 | 17156 | 8780816 | 3506984 | 13.32x |
| M28_2019spring_19_2 | 5729992 | 54601 | 12455 | 5235037 | 2264194 | 10.25x |
| M28_2019spring_19_3 | 8290564 | 58559 | 15725 | 7599564 | 3406862 | 13.20x |
| M28_2019spring_2_1 | 8727812 | 49925 | 13705 | 8027336 | 2805538 | 12.47x |
| M28_2019spring_20_1 | 17048598 | 103944 | 27257 | 15924001 | 5867274 | 19.38x |
| M28_2019spring_20_2 | 22648814 | 82847 | 24346 | 21467095 | 3595604 | 13.92x |
| M28_2019spring_3_1 | 7425328 | 82102 | 18152 | 6593760 | 3357092 | 12.66x |
| M28_2019spring_3_2 | 7022160 | 50586 | 10956 | 6507895 | 2854534 | 11.80x |
| M28_2019spring_4_1 | 7170644 | 98207 | 30728 | 6166731 | 3019550 | 11.80x |
| M28_2019spring_5_1 | 17778348 | 71506 | 23444 | 16659281 | 1663802 | 7.01x |
| M28_2019spring_5_2 | 8221128 | 81683 | 19731 | 7442476 | 3897926 | 14.43x |
| M28_2019spring_5_3 | 18445174 | 96367 | 27355 | 17014932 | 2465334 | 8.54x |
| M28_2019spring_6_2 | 8304112 | 51987 | 16243 | 7605455 | 3677910 | 13.63x |
| M28_2019spring_6_3 | 44355008 | 104638 | 42153 | 41818223 | 1481542 | 6.06x |
| M28_2019spring_7_1 | 8252248 | 68669 | 12340 | 7653000 | 3579244 | 14.15x |
| M28_2019spring_8_2 | 10123398 | 51282 | 16860 | 9311661 | 3980146 | 14.91x |
| M28_2019spring_9_2 | 6234686 | 70889 | 15895 | 5577063 | 2819820 | 12.65x |
| M28_2019spring_9_3 | 20371992 | 76316 | 21124 | 18856637 | 2048688 | 8.84x |

Appendix Figures:

Appendix Fig. 1. Distribution of GC content across 1.6 million RAD loci found by the Stacks *de novo* pipeline using default parameters (a). The histogram shows a secondary GC peak likely representing bacterial contamination. RAD loci were aligned to the NCBI *nt* database with *blastn* with an E-value cutoff set to 1e-05. Any locus with a hit to non-cnidarian sequences was considered a contaminant and filtered out to form a contaminants database. Without these contaminants the secondary GC peak was substantially lower (b). The top hits were to *Anthoathecata* (the cnidarian order to which *Hydra* belongs), three bacterial orders (*Burkholderiales*, *Pseudomonadals* and *Aeromonadales*) and *Anostraca* (most likely originating from hydra food).


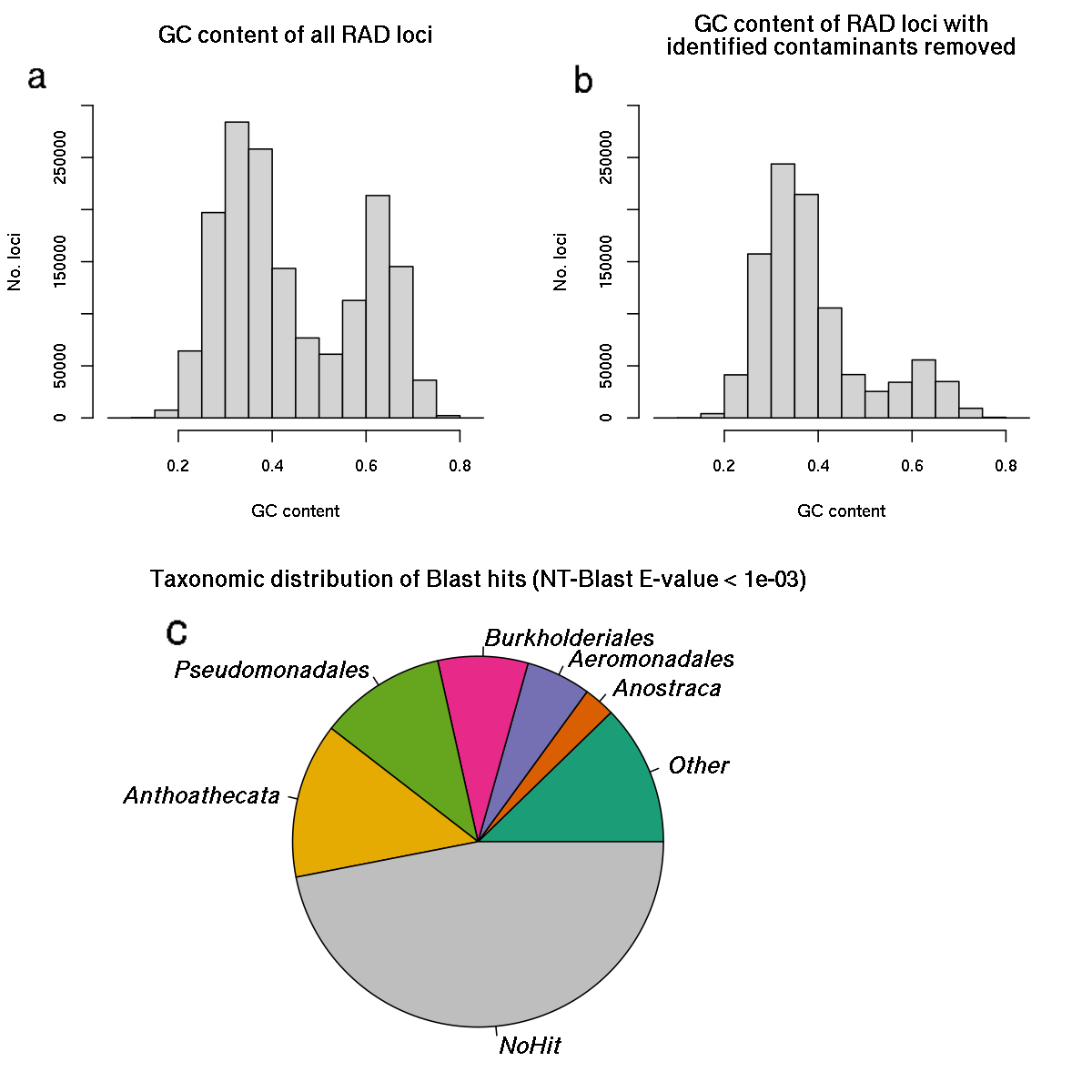


Appendix Fig. 2. Spectrum of genetic diversity of N=132 H. oligactis strains showing a clear peak of low genetic relatedness (<~0.06, supposed clones) and a secondary peak ~0.11, which can belong to other multilineage genotypes assuming a high genotyping error rate / somatic mutation rate, or distinct multilineage genotypes.


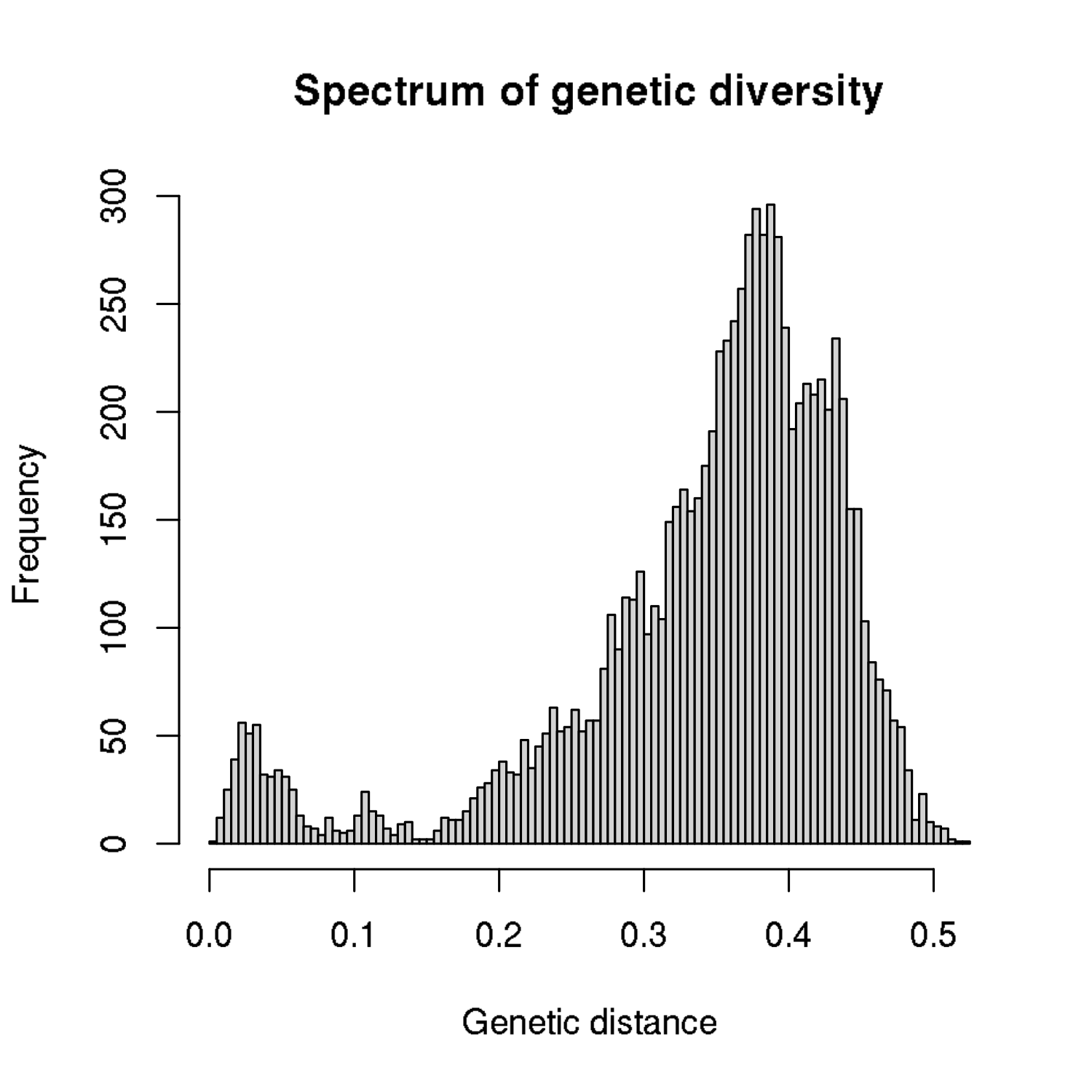

Supplement: Supplementary file 1 — Appendix S1 [file ECE3-12-e9096-s001.docx]
